# Scramble-Free Synthesis of Unhindered *trans*-A_2_B_2_-Mesoaryl Porphyrins via Bromophenyl Dipyrromethanes

**DOI:** 10.1021/acs.orglett.3c04215

**Published:** 2024-02-19

**Authors:** Muteb H. Alshammari, Sultanah M. N. Alhunayhin, David L. Hughes, Isabelle Chambrier, Andrew N. Cammidge

**Affiliations:** School of Chemistry, University of East Anglia, Norwich Research Park, Norwich NR4 7TJ, U.K.

## Abstract

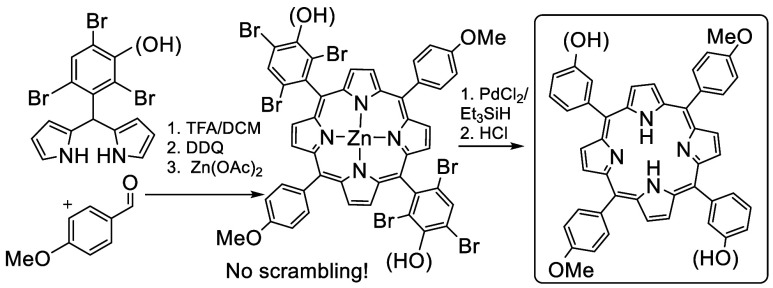

*Trans*-disubstituted porphyrins are highly valuable intermediates across diverse fields, but they pose a significant synthesis challenge in some cases due to scrambling and formation of complex mixtures. Conditions that minimize scrambling also lower yields, but steric hindrance around the meso-aryl substituent can effectively suppress scrambling altogether. Here we report a straightforward approach to valuable trans-A_2_B_2_ porphyrin intermediates that are otherwise very difficult to obtain, through use of removable blocking bromide substituents.

The synthetic chemistry of porphyrins was first revolutionized by Adler and Longo’s simple procedure that permitted easy access to *meso*-aryl porphyrins in a single step from pyrrole and aromatic aldehydes by refluxing in propionic acid open to air.^1^ The general methodology was subsequently refined and expanded by Lindsey and co-workers who, among many other developments, introduced higher yielding protocols that employed milder acidic conditions in organic solvents to allow the incorporation of more sensitive substrates (Scheme 1).^2^

**Scheme 1:**
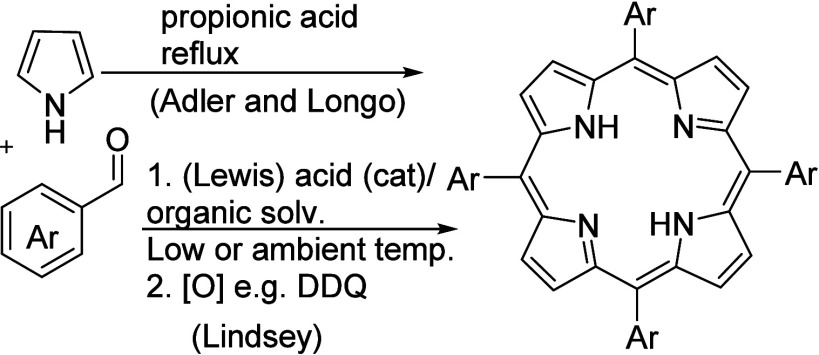
Direct Synthesis of *meso*-Aryl Porphyrins

Unsymmetrically substituted derivatives can be accessed through a mixed condensation of pyrrole with two different aldehydes. As expected, reactions of this type produce a complex mixture with low yields of the individual products isolated after challenging separations. The reactions can be useful for synthesis of A_3_B type porphyrins, and we have exploited this in our own work for building symmetrical diporphyrins as precursors to multidecker systems,^3^ and unsymmetrical chromophore dyads.^4^ The strategy is rarely useful for A_2_B_2_ derivatives where both AABB (*cis*) and ABAB (*trans*) isomers are formed. The *trans* isomers are highly valued intermediates and have been widely employed across diverse fields including supra-/supermolecule construction and catalysis.^5−7^ A rational approach to the synthesis of *trans*-ABAB porphyrins exists^6^ whereby a preformed dipyrromethane is condensed with a different aldehyde. The synthesis, which follows from MacDonald’s original use of a dipyrromethane dialdehyde + dipyrromethane,^8^ is widely employed and is highly successful in selected cases. However, a major problem that is inevitably encountered in these syntheses is that of scrambling, whereby acidolysis of dipyrromethane and/or higher oligomers (essentially reverse condensation) leads to a set of products identical to that expected from a simple mixed condensation with two aldehydes. The reaction has been carefully and systematically investigated, and conditions have been developed to minimize scrambling. Typically, conditions that minimize scrambling have a negative impact on yield, but a key observation is that significant steric hindrance around the *meso*-aryl substituent can effectively suppress scrambling altogether (Scheme 2).^6^ In many cases this is a benefit because the same bulky substituents aid the porphyrin solubility (useful in supra- and supermolecule construction and characterization) and can affect the environment above and below the porphyrin plane around the axial position of any incorporated metal ion, a feature that can be exploited in catalysis.^9^

**Scheme 2:**
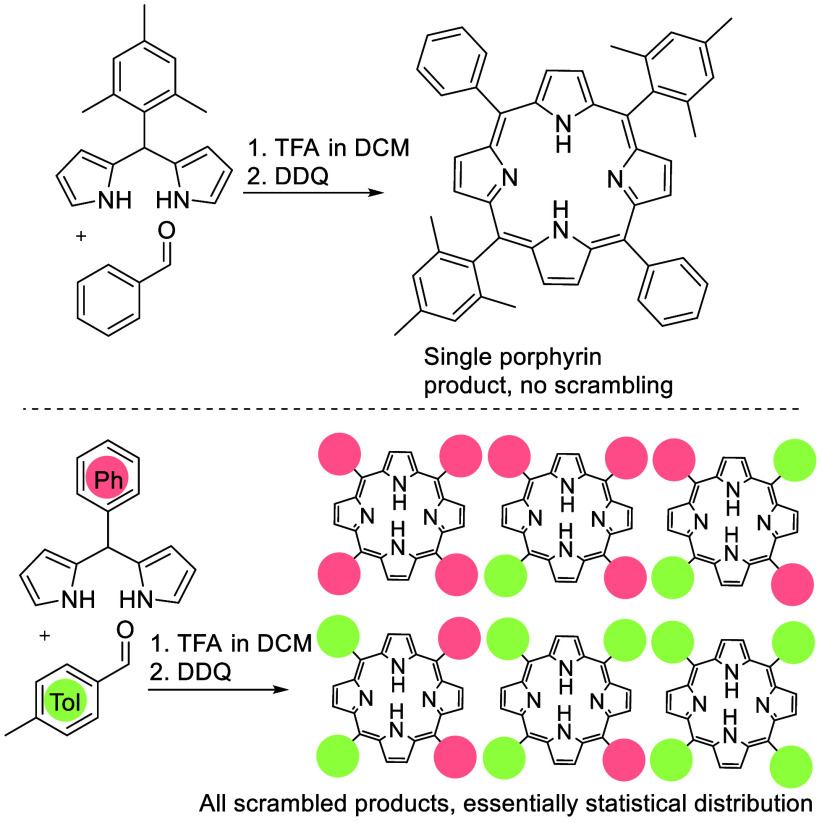
Efficient Rational Synthesis of *transA*_*2*_*B*_*2*_*meso*-Aryl Porphyrins Using a Dipyrromethane Bearing a Sterically Hindered Aryl (e.g., Mesityl, Top) and Inefficient Synthesis Due to Scrambling When Unhindered Aryl Substituents Are Employed (Bottom)^6b^

In our ongoing work on heteroleptic triple-decker porphyrin–phthalocyanine complexes^3^ we required efficient syntheses of differentially substituted *trans* ABAB-*meso*-aryl porphyrins suitable for further elaboration at either of the 5,15-positions only, or separately at the 5,15- and then 10,20-positions. The planned chemistry is one example where the use of sterically hindered aryl substituents cannot be used, because the hindrance required for efficient *trans*-porphyrin synthesis prevents the subsequent face-to-face assembly of multidecker complexes. Here even fluorine substituents on the 2,6-positions prevent face-to-face assembly and essentially only hydrogen can be accommodated. However, porphyrins bearing only remote functionality (3- and 4-positions) are valuable for elaboration in many other areas also, for example to build oligomers and polymers, and for attachment to complementary organic and inorganic species and surfaces.^10^ We particularly targeted *trans*-porphyrins bearing opposite pairs of hydroxyl and/or methoxy groups, knowing the latter can be selectively hydrolyzed to reveal phenolic residues following alkylation of the first pair of phenols and therefore provide valuable versatility for further stepwise elaboration. *trans*-Bis(4-methoxyphenyl)porphyrin **2** can be prepared using the dipyrromethane route, but the outcome is similar to the standard mixed porphyrin synthesis from aldehyde precursors. The reactions yielded the full mixture of scrambled products from which the dimethoxy isomeric mixture can be isolated in low (5–12%) yield (Scheme 3). The isomers cannot be separated, but NMR analysis of the mixture shows that the ratio of isomers is between 2:1 and 1:1 (Supporting Information). Hydrolysis allows careful separation of the isomeric diols and reveals the major isomer to be *cis* (5,10-). The presence of the activating methoxy substituent no doubt accelerates acidolysis. The reason for domination of the *cis*-isomer over *trans* is less clear, but the result is consistent with reported direct synthesis of di(4-hydroxyphenyl)porphyrins where the *cis*-isomer is also formed preferentially.^11^ The differentially substituted *trans* di(4-methoxyphenyl)-di(3-hydroxyphenyl)porphyrin **4** is unknown, and there is no obvious direct synthesis possible. Our brief investigation of mixed cyclizations confirmed that separation of the porphyrin mixture, and particularly the isomers, would be impractical.

**Scheme 3:**
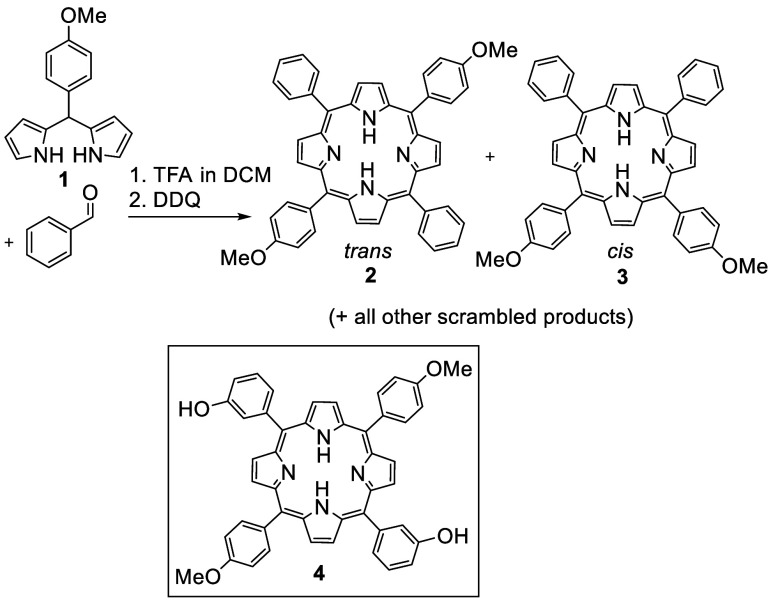
Attempted Synthesis of *trans*-Dimethoxyphenyl Porphyrin **2** Is Inefficient Due to Scrambling and Favors *cis*-Isomer Production (Top); Lightly Functionalized Opposite-Opposite *trans*-Substituted Porphyrins Like the Target **4** (Bottom) Are Not Accessible

While multistep synthesis via cross-coupling strategies is possible,^12^ we reasoned that the most pragmatic solution to overcoming the scrambling issue would be to employ steric blocking groups that could later be removed after guiding efficient dipyromethane + aldehyde porphyrin synthesis without scrambling. The success of the sequence would rely on the ready availability of suitable precursors, so that the overall effectiveness of the sequence outweighed the inherent low atom economy of protecting group strategies. A survey of suitably designed benzaldehyde derivatives highlighted the potential of 3-hydroxy-2,4,6-tribromobenzaldehyde **5** which is readily available both commercially and from bromination of 3-hydroxybenzaldehyde.^13^ We recognized that aldehyde **5** could act as a precursor for both the complex, differentially substituted porphyrin **4**, and the simple (but difficult to access) *trans*-dimethoxyporphyrin **2** via common intermediate *trans*-porphyrin **7**. The sequence is shown in Scheme 4 along with the simple statistical porphyrin synthesis that was employed to generate a suitable model porphyrin **9** for the initial evaluation of deprotection (reduction) conditions.

**Scheme 4:**
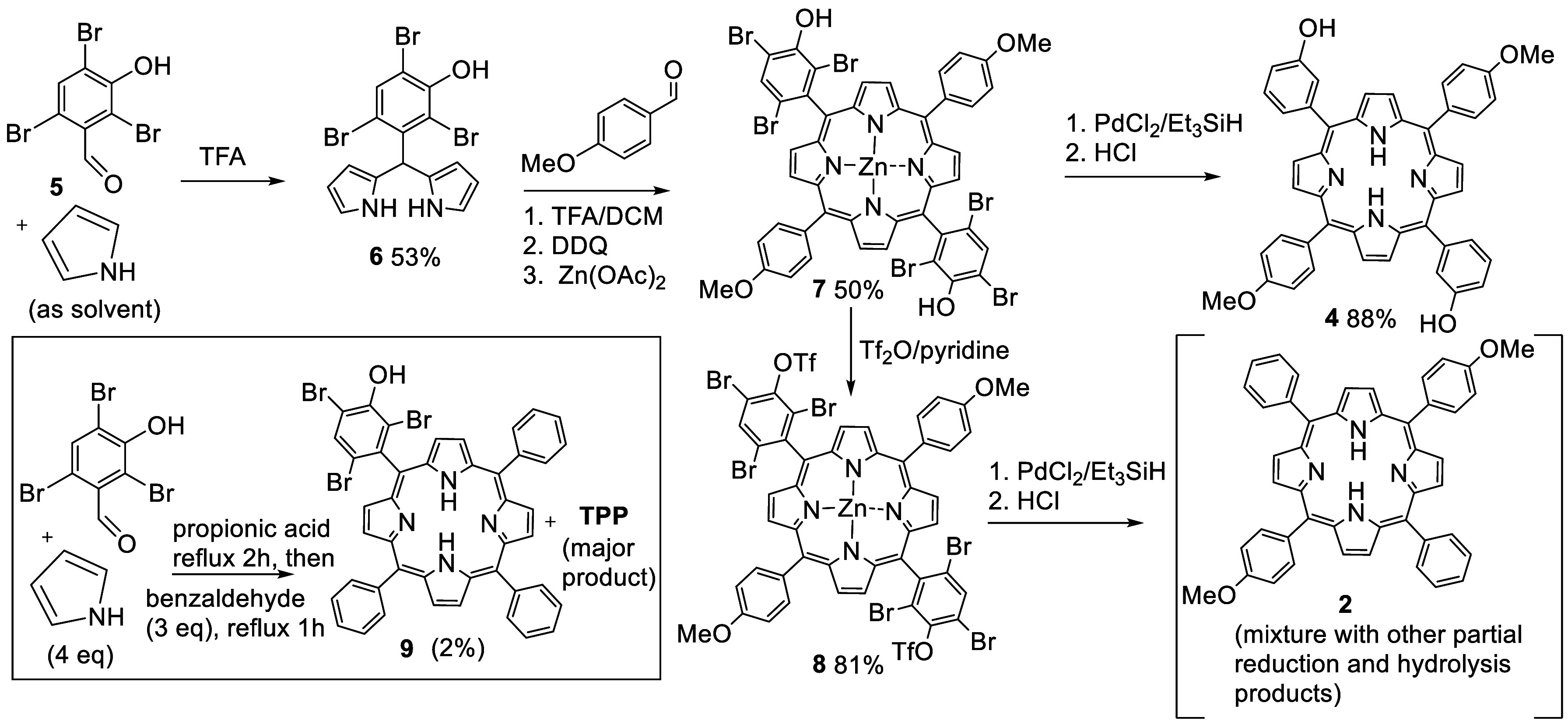
Synthesis of Lightly Substituted *Trans* Porphyrins Employing Removable Bromide Substituents To Prevent Scrambling

Unsurprisingly the hindered aldehyde **5** proved to be less reactive than benzaldehyde itself, resulting in a 2% yield of the 3:1 porphyrin **9** alongside tetraphenylporphyrin (TPP) as the major porphyrin product. Nevertheless, sufficient porphyrin **9** was isolated to allow investigation of known reduction conditions employing triethylsilane and palladium chloride catalyst (Scheme 5).^14^ Porphyrin **9** and PdCl_2_ (5 mol %) were heated in triethylsilane at 120 °C in a sealed tube, and the reaction was monitored periodically by analysis of aliquots by MALDI-MS. Reduction proceeded slowly, and it was clear that palladium porphyrin derivatives were also formed in the process. While this is not surprising, and the palladium can be easily removed in the acidic workup, the side reaction effectively removes the palladium catalyst from the system and slows the rate. Nevertheless, mono-(3-hydroxyphenyl)porphyrin **10** was isolated after workup (HCl) in 62% yield.

**Scheme 5:**
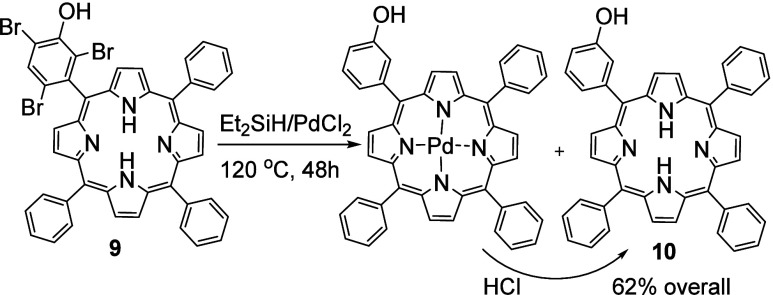
Reductive Debromination of Porphyrin **9** Using Triethyl Silane and Palladium Chloride

The main synthesis of *trans*-porphyrin targets began with straightforward synthesis of dipyrromethane **6** from the reaction of aldehyde **5** with excess pyrrole (used as reactant and solvent). As expected, dipyrromethane **6** proved to be relatively stable and could be stored for several weeks as a crystalline solid in the dark at 0–5 °C without any noticeable degradation. Pleasingly the synthesis of the corresponding *trans*-porphyrin was also smoothly achieved using 4-methoxybenzaldehyde following conditions developed by Lindsey,^2^ and it is worth noting this electron-rich, unhindered aldehyde represents one of the most challenging reactants in terms of suppressing scrambling during porphyrin synthesis. However, based on the previous observations during reductive debromination of the model porphyrin **9**, we decided to insert zinc at the end of the reaction in order to prevent palladium sequestration during subsequent reduction. Dipyrromethane **6** and 4-methoxybenzaldehyde were therefore reacted together in DCM (0.85 mM) with TFA catalyst at 0 °C. At the end of the reaction, DDQ was added followed (after 1 h) by addition of Zn(OAc)_2_. *Trans* porphyrin **7** was isolated as the only observed porphyrin product in a 50% yield. Porphyrin **7** exists as an equilibrated mixture of atropisomers. They appear as two distinct spots by tlc but are essentially identical in ^1^H NMR spectroscopy. Atropisomer interconversion occurs in minutes at room temperature (tlc).

Reductive debromination of zinc porphyrin **7** was achieved smoothly by using triethylsilane and PdCl_2_ at 120 °C for 3–5 days. The crude reaction mixture was treated with HCl to remove zinc, neutralized, and separated to give the target differentially substituted *trans* porphyrin **4** in 88% yield. Alternative reduction conditions using formate and palladium, successfully employed by us in other projects for reduction of Ar–X,^15^ gave very slow reduction, an observation that likely reflects the effect of the electron-donating hydroxyl group in retarding palladium insertion (oxidative addition) into the Ar–Br bonds.

Conversion of intermediate **7** into *trans* di(methoxyphenyl) porphyrin **2** required reductive removal of both the bromide and hydroxyl substituents and was achieved by first converting the free phenols to triflates using triflic anhydride. Reduction of triflate **8** was attempted by using both PdCl_2_/Et_3_SiH and Pd/formate conditions. In each case the reduction of both triflate and bromide could be achieved, but the reactions were slow and impractical. Each reaction was monitored periodically by MALDI-MS. Under PdCl_2_/Et_3_SiH conditions, the MALDI-MS spectra clearly demonstrated initial preferential (faster) removal of bromides but very sluggish aryl triflate reduction (incomplete after 7 days at 120 °C). Reductions using Pd/formate conditions were also inefficient and slow, with evidence for competing triflate hydrolysis at long reaction times. Rather than pursue investigation of alternative conditions to achieve this, more simple, target, we elected to instead use the developed blocking strategy with an alternative bromoaryl aldehyde. Fortunately, 2,6-dibromobenzaldehyde is available. Indeed, it has been previously employed for synthesis of *trans* porphyrins, although not with challenging electron-rich partners.^9^ The convenient synthesis of *trans* di(methoxyphenyl) porphyrin **2** is shown in Scheme 6.

**Scheme 6:**
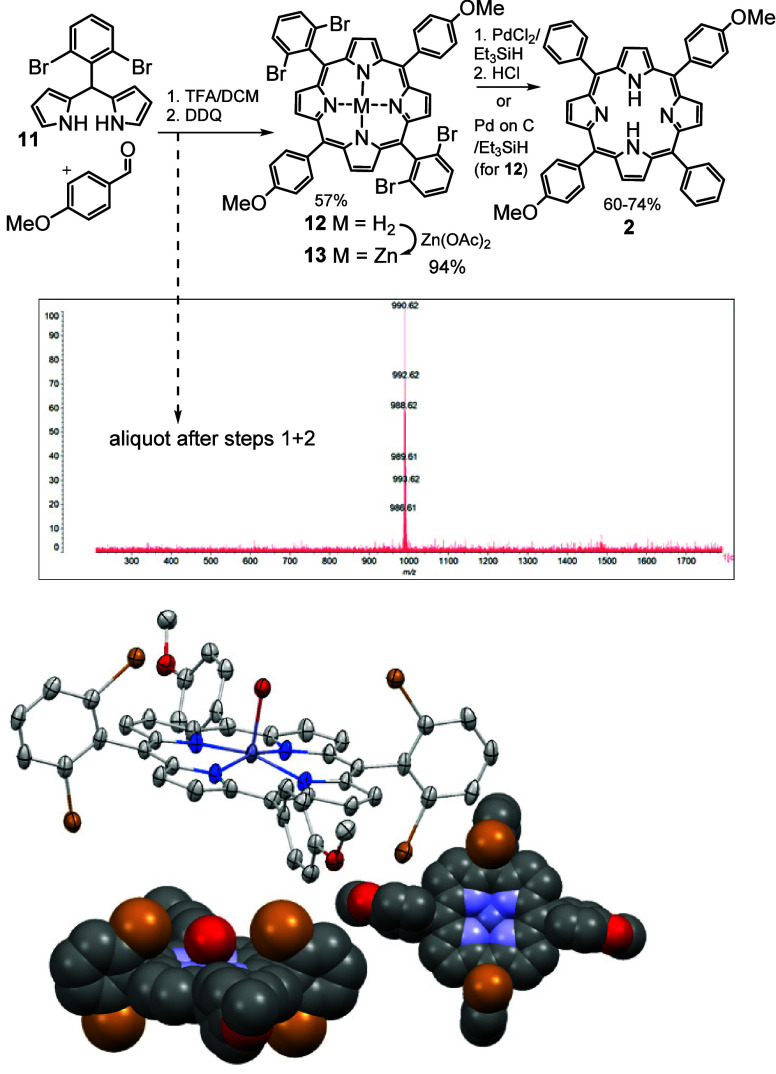
Convenient Synthesis of *trans* Dimethoxyporphyrin **2** (Inset Shows the Single Mass Observed in MALDI-MS of a Sample before Addition of Zinc Acetate), and the X-ray Crystal Structure for Porphyrin **13** (Shown as Elipsoids at 65% Probability, H-Atoms and Molecule of Chloroform Removed for Clarity)

Dipyrromethane **11** was synthesized as reported,^9^ and reaction first with 4-methoxybenzaldehyde, then DDQ, and then zinc acetate gave the corresponding *trans* Zn-porphyrin **13** with no evidence for other scrambled products; Scheme 6 (inset) shows the MALDI-MS taken for an aliquot of the reaction mixture, before addition of zinc acetate, with essentially a single signal (cluster at *m*/*z* 990.62) corresponding to porphyrin **12**. The zinc porphyrin **13** showed a strong tendency toward crystallization that complicated chromatographic purification. It was found to be more convenient to first isolate the metal-free porphyrin **12** (57%) and then insert zinc in a separate step (94%). Crystals of porphyrin **13** suitable for X-ray diffraction were obtained (CCDC 2313839), and the structure is also shown in Scheme 6. The space-filling representations clearly illustrate the effective steric blocking of *meso*-sites provided by the *o*-bromines. Reduction also proceeded smoothly using PdCl_2_/Et_3_SIH and gave the desired porphyrin **2** in 74% yield after workup (HCl) and straightforward isolation. Direct reductive debromination of metal-free *trans* porphyrin **12** was also investigated by using palladium on carbon. Pleasingly the reduction works well, again employing triethylsilane, giving porphyrin **2** in 60% yield.

In conclusion, we have developed a straightforward approach to valuable *trans*-A_2_B_2_ porphyrin intermediates that are otherwise very difficult to obtain by direct methods. Access to such porphyrins, which bear remote functionality but lack excessive steric blocking on the porphyrin core, opens the potential for wide application, particularly in super/supramolecule construction and surface grafting.

## Data Availability

The data underlying this study are available in the published article and its Supporting Information.

## Supporting Information Available

Experimental and characterization data for synthesized compounds plus crystallography details for porphyrin **13** (PDF)
