# Supplementary material for: Scramble-Free Synthesis of Unhindered trans-A2B2-Mesoaryl Porphyrins via Bromophenyl Dipyrromethanes
Source: Org Lett. 2024 Feb 19;26(8):1561–5. doi: 10.1021/acs.orglett.3c04215 (PMC10913071; doi:10.1021/acs.orglett.3c04215)
Supplement: Supplementary file 1 — ol3c04215_si_001.pdf [file ol3c04215_si_001.pdf]

Scramble-free synthesis of unhindered *trans*-A_2B_2-mesoaryl porphyrins via bromophenyl dipyrromethanes.

Muteb H. Alshammari, Sultanah M. N. Alhunayhin, David L. Hughes, Isabelle Chambrier and Andrew N. Cammidge*

School of Chemistry, University of East Anglia, Norwich Research Park, Norwich NR4 7TJ, UK.

Corresponding author email address: a.cammidge@uea.ac.uk

Contents

General information……………………………………………………………………………2

Experimental part…………………………………………………………………………………3

Crystallography details for porphyrin 13 …………………………………………..21

General Information

Silica gel (Material Harvest or Merck) 40-63 micron was used for column chromatography. Petroleum ether refers to boiling range 40-60 ^{o}C. A rotary evaporator was used to evaporate the solvents and the process was carried out at reduced pressure. NMR spectra (^{1}H NMR at 500 MHz, ^{13}C NMR 125.7 MHz were recorded on a Bruker Ascend 500 instrument; ^{1}H NMR at 400 MHz, ^{13}C NMR 100.6 MHz were recorded on Ultrashield Plus^{TM} 400 instrument) and the residual solvent peak was used as reference. Spectra were recorded at room temperature. ^{13}C spectra could not be obtained for some porphyrins due to low solubility. A Shimadzu Biotech Axima spectrometer was used to record the MALDI-TOF MS analyses and matched to theoretical isotopic distribution patterns to confirm the assigned molecular ion. UV-vis spectra were measured in the stated solvents using a Hitachi U-3310 instrument.

Experimental part:

5-(4-Methoxyphenyl)dipyrromethane (1)[1]

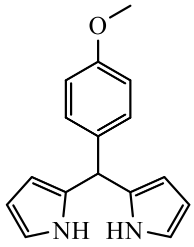

A mixture of 4-methoxybenzaldehyde (1.96 g, 1.75 ml, 14.4 mmol) and freshly distilled pyrrole (24.1 g, 25 ml, 360 mmol) was degassed by bubbling argon for 30 min. Trifluoroacetic acid (0.11 ml, 0.10 eq) was added to the solution and stirring continued under Ar at room temperate for 5 min. NaOH (0.1 M) was used to quench the reaction then ethyl acetate was added. The filtrate was washed with water and the organic layer dried over Na_2SO_4. The solvent and unreacted pyrrole were further removed under vacuum at 60 °C. The resulting oil was then purified by column chromatography using DCM/Pet ether (1.5:1) and recrystallization from ethyl acetate/hexane to yield the product as off-white crystals (2.23g, 61%) **^{1}H NMR (400 MHz, Chloroform-*d*)** δ 7.90 (br-s, 2H), 7.14 (d, *J* = 8.5, 2H), 6.86 (d, *J* = 8.5, 2H,), 6.70 (ddd, *J* = 2.9, 2.7, 1.6 Hz, 2H), 6.16 (dd, *J* = 2.9, 2.7 Hz, 2H), 5.93-5.90 (m, 2H), 5.44 (s, 1H), 3.80 (s, 3H).

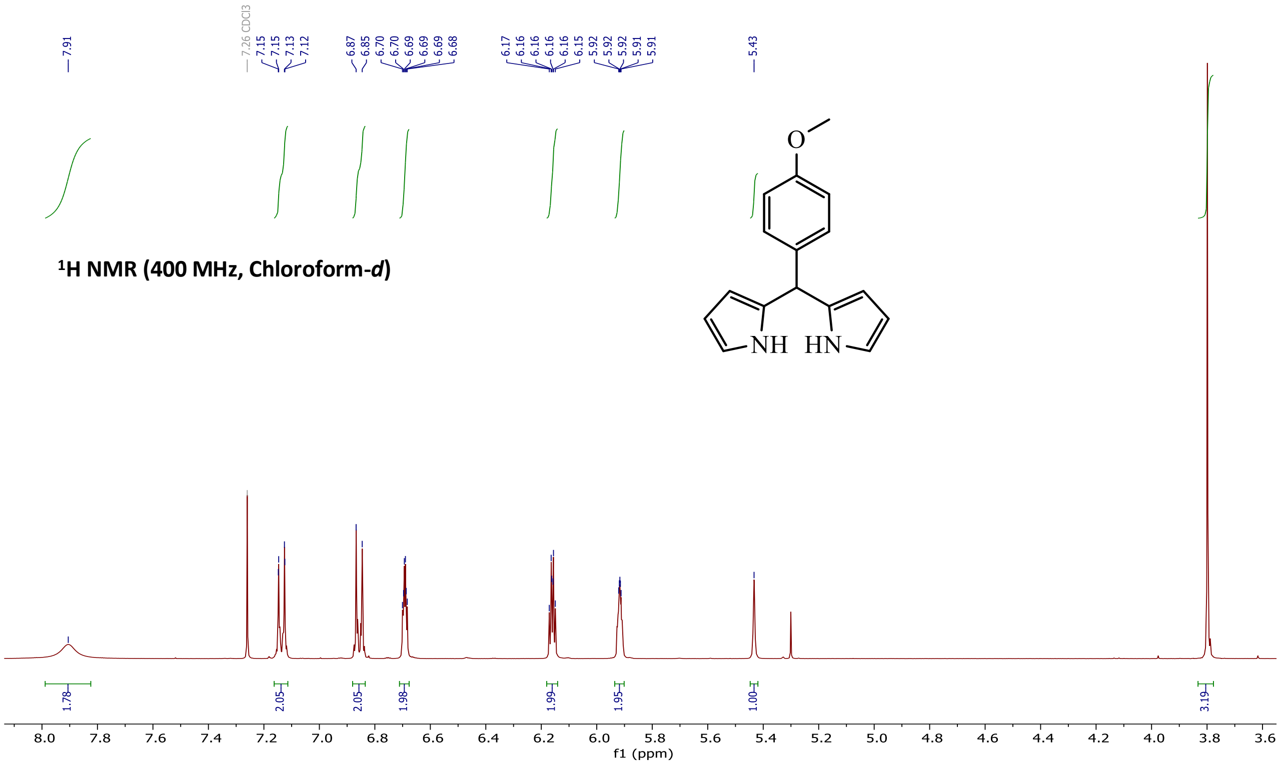

5,15-Diphenyl-10,20-bis(4-methoxyphenyl)porphyrins (2+3)

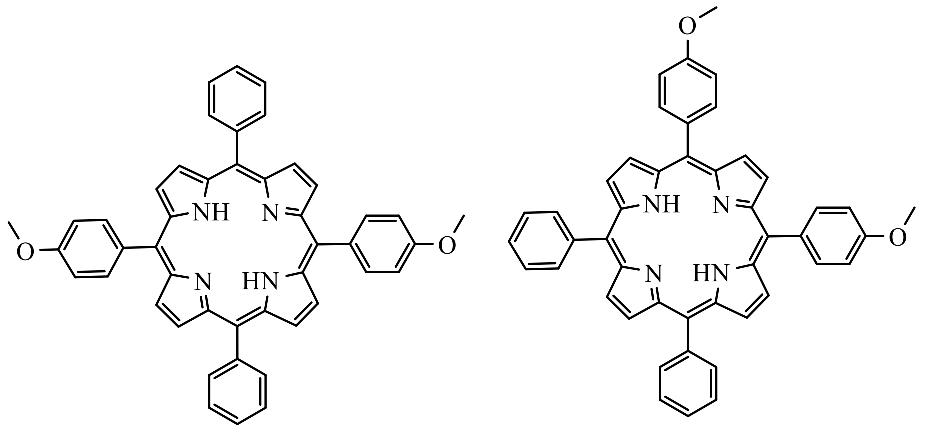

Method A:

4-Methoxydipyrromethane **1** (2.52 g, 10 mmol) and benzaldehyde (1.06 g, 10 mmol) were dissolved in anhydrous DCM (1000 ml) and the reaction mixture was stirred under nitrogen. TFA (1.37, 17.8 mmol) was added slowly to the mixture at room temperature and the solution was stirred at room temperture for 1.5h. DDQ (2.27 g, 10 mmol) was added, and the mixture was stirred for 60 min. The crude solution was poured through an alumina pad and eluented with DCM until the solution became brown. The solvent was removed to give a dark solid which was dissolved in toluene with DDQ and heated to reflux for further 1 h to remove any remaining porphyrinogen. After cooling to room temperature, the solvent was removed, and column chromatography was used to isolate the desired product using DCM/Pet ether (2:1) as an eluent. The third fraction was the mixture of *cis* and *trans* isomers porphyrin (~2:1, 0.4g, 12%).

Method B:

Following the Adler method, 4-methoxybenzaldehyde (6.8 g, 50 mmol) and benzaldehyde (5.3 g, 50 mmol) were dissolved and heated at reflux in propionic acid (200ml). After the mixture started refluxing, distilled pyrrole (6.7 ml, 6.5 g, 100 mmol) was added dropwise and left for 30 min. After cooling down to ambient temperature, methanol (150 mL) was added, and the mixture was left in a fridge to precipitate. The purple solids were collected by vacuum filtration, then purified by column chromatography using DCM/Pet ether (2:1) as an eluent. The mixture of *cis/trans* isomers were collected as a third purple fraction (~2:1, 0.85 g, 5 %). **^{1}H NMR (400 MHz, Chloroform-d)** δ 8.87 (d, J = 3.1 Hz, 4H), 8.84 (d, J = 3.3 Hz, 4H), 8.22 (d, J = 6.0 Hz, 4H), 8.13 (d, J = 8.7 Hz, 4H), 7.83 – 7.70 (m, 6H), 7.29 (d, J = 8.8 Hz, 4H), 4.10 (s, 6H), -2.76 (s, 2H).

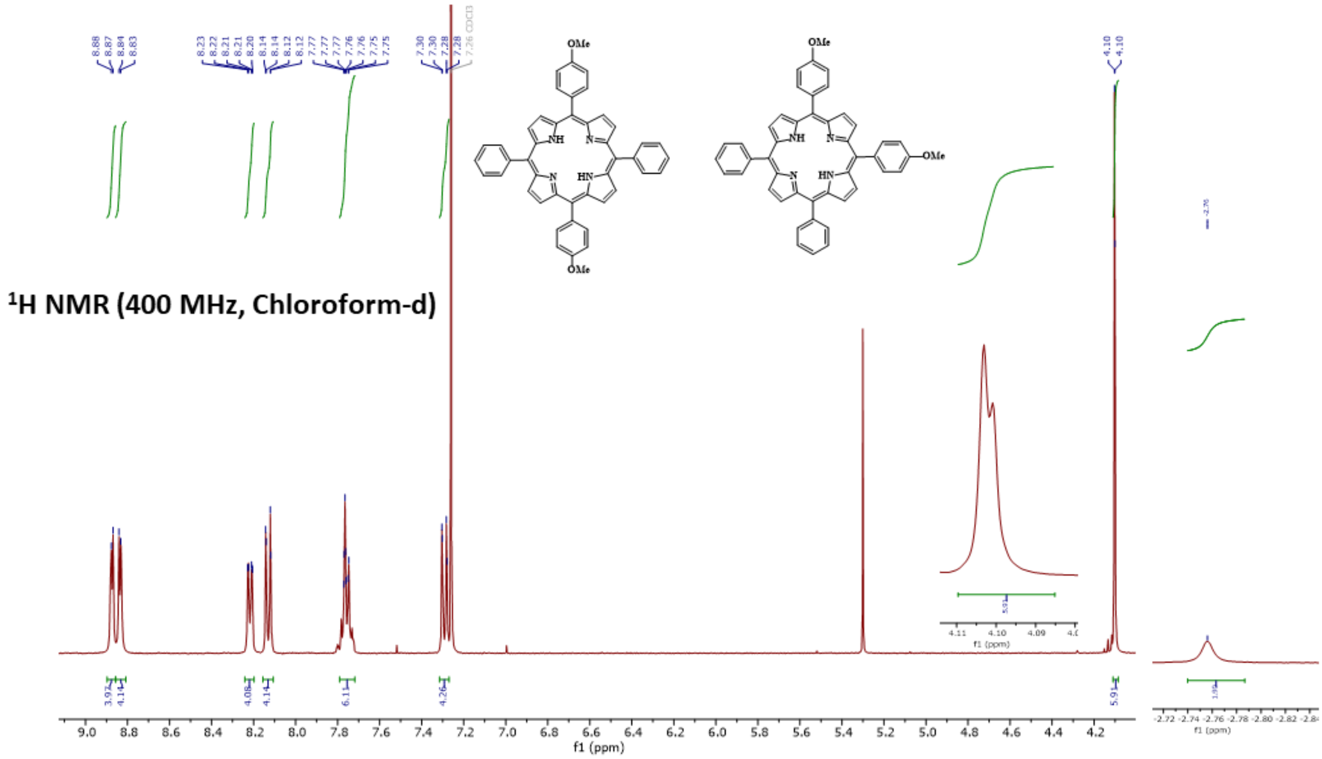

2,4,6-tribromo-(3-hydroxyphenyl)-porphyrin) (9)

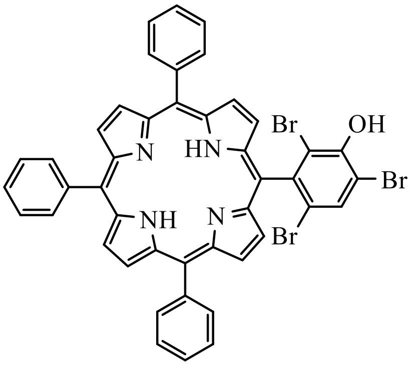

2,4,6-Tribromo-3-hydroxybenzaldehyde **5** (2g, 5.60 mmol) and freshly distilled pyrrole (1.5g, 22.4mmol) were mixed in propionic acid (150 mL) then the mixture was heated to reflux for 2h. benzaldehyde (1.8g, 16.8mmol) was then added dropwise. After the addition was finished, the resulting mixture was refluxed for a further hour. The mixture was cooled down to room temperature. MeOH (200 mL) was added, then left overnight t 5 ^{o}C to precipitate. The resulting purple solid was filtered off and washed with MeOH. The crude compound was purified by chromatography using DCM: PET (1:3 v: v) as eluent. After collecting all the first fraction (symmetrical tetraphenyl porphyrin, TPP) the polarity of the eluent was increased to 100% DCM to collect the product as the second dark purple fraction. The compound was recrystallized from DCM:MeOH to give 2,4,6-tribromo-(3-hydroxyphenyl)porphyrin **9** as a purple solid (55 mg, 2%) that was used in the model debromination experiments without further purification. **^{1}H NMR (500 MHz, Methylene Chloride-*d*$_2$)** δ 8.83-8.69 (m, 8H), 8.18 – 8.06 (m, 8H), 7.78 – 7.63 (m, 8H), -2.84 (2 x br s, 2H) NH; **MS (MALDI-tof) m/z:** [M$^+$] Calcd for $C_{44}H_{27}Br_3N_4O$ 866; Found 866 and

868 (cluster); **UV-vis, λmax (DCM)/nm** 418, ε=5.12x10^5 ; 515, ε=2.17x10^4 ; 548, ε=8.02x10^3 ; 597, ε=5.09x10^3 ; 651, ε=4.32x10^3.

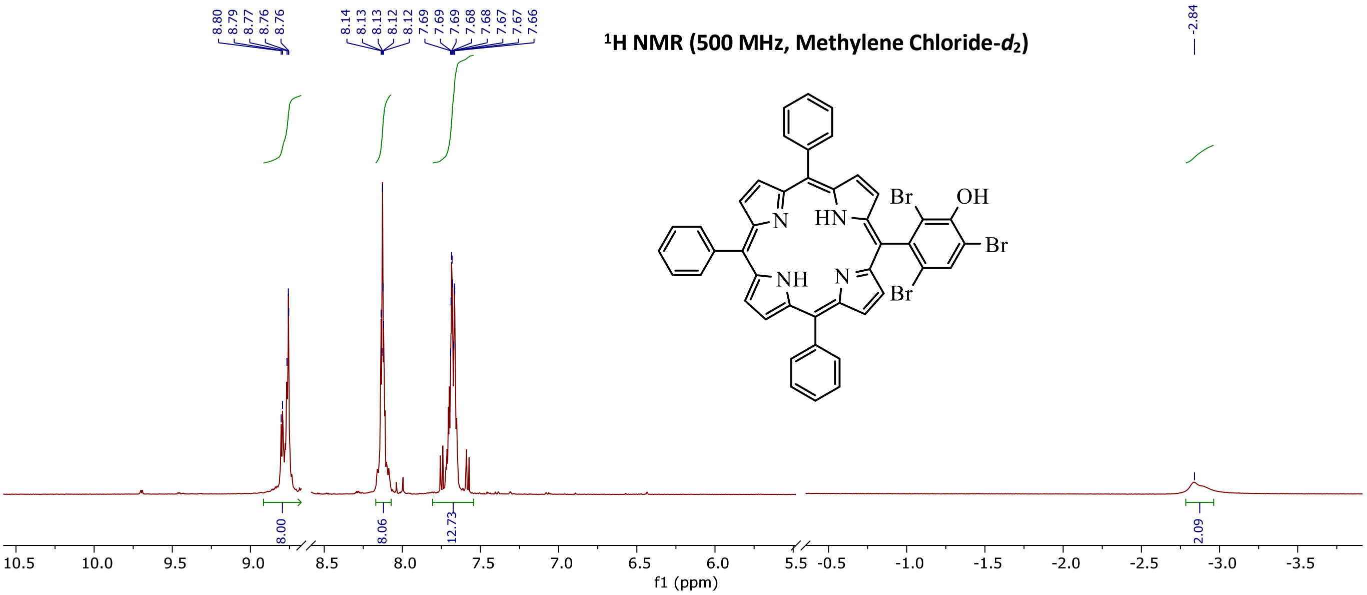

5,10,20-(triphenyl)-15-(3-hydroxyphenyl)porphyrin (10)[2]

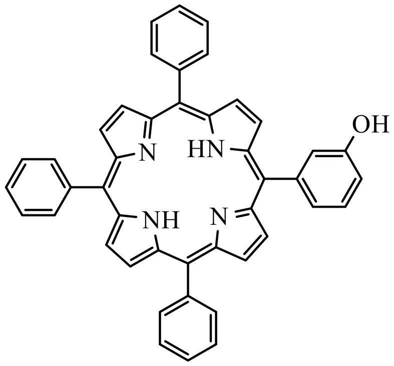

Tribromoporphyrin **9** (30 mg, 0.035 mmol) was dissolved in triethylsilane (4 ml) and palladium chloride (ca 0.5 mg, ~5 mol%) was added as catalyst under argon in a sealed tube. The reaction was heated at 120°C for 3 days. The mixture was cooled to room temperature and then the solvent was evaporated under reduced pressure. The resulting product, a green solid, was treated with several drops of concentrated HCl to remove any Pd which had inserted into the porphyrin. The crude compound was further worked up by addition of water and TEA (1 ml) then extracted with DCM (3x100 ml), dried over anhydrous $MgSO_4$ and the solvent was evaporated under reduced pressure to give a crude product which was purified using column chromatography on silica gel with DCM and recrystallized from DCM/PET (1:1) to obtain porphyrin **10** as a purple solid (13 mg, 62 %).

^{1}H NMR (500 MHz, Chloroform-*d*) δ 8.89 (d, *J* = 4.8 Hz, 2H), 8.86 – 8.82 (m, 6H), 8.25-8.19 (m, 6H), 7.81 – 7.73 (m, 9H), 7.70-7.68 (m, 1H), 7.52 (t, *J* = 8.3, 1H), 7.27-7.12 (m, 1H), -2.86 (s, 2H) NH.

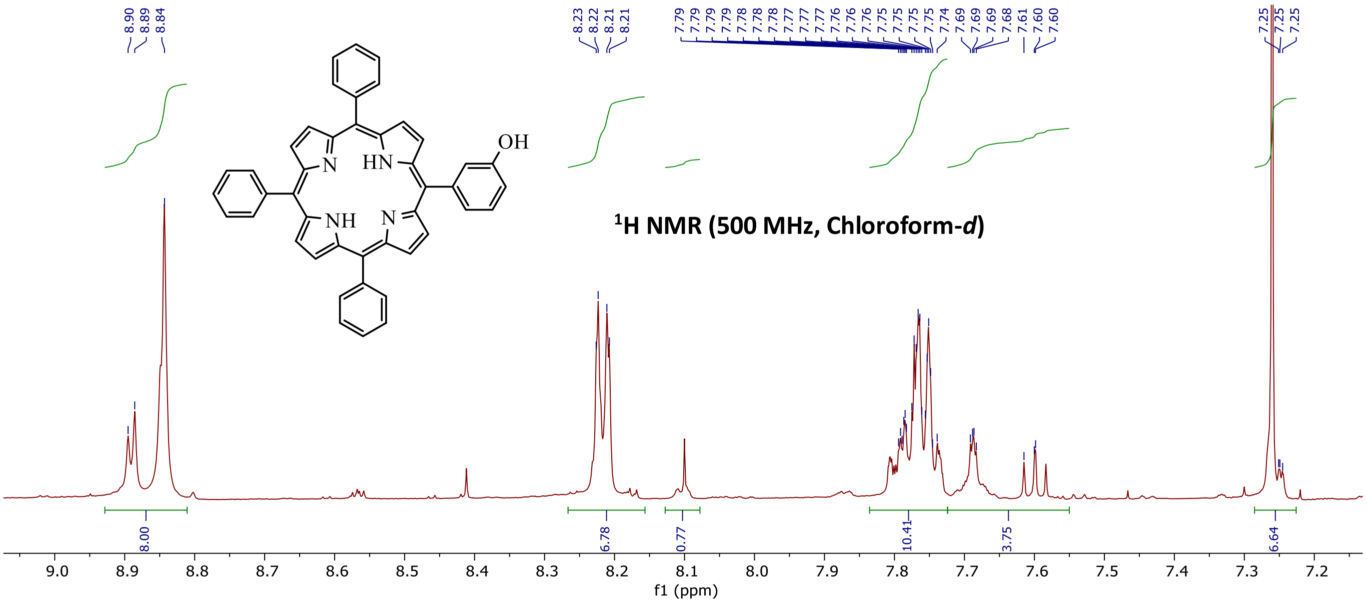

(2,4,6-Tribromo-5-hydroxyphenyl)dipyrromethane (DPM) (6)

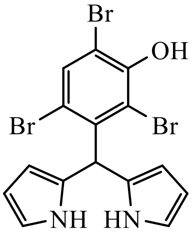

A mixture of 2,4,6-tribromo-5-hydroxybenzaldehyde (2 g, 5.57 mmol) and freshly distilled pyrrole (9.38 g, 9.70 ml, 140 mmol) were stirred at room temperature under argon for 20 min. Trifluoroacetic acid (0.04 ml, 0.56 mmol) was added slowly to the mixture and left stirring for 5 min. NaOH (0.1 M) was added to quench the reaction which was then extracted with ethyl acetate. The organic layer was washed with water (x3) and dried over anhydrous Na_2SO_4. The solvent was removed under reduced pressure to give a green oil which was chromatographed (silica gel, DCM 100%). Crystallization from DCM/Pet ether afforded DPM **6** as pale yellow crystals (1.4 g, 53%). **Mp** 185-187 ^{0}C; **^{1}H NMR (400 MHz, Chloroform-d)** δ 8.26 (br-s, 2H) 7.76 (s, 1H) , 6.74 (ddd, J = 2.9, 2.7, 1.5 Hz, 2H), 6.47 (s, 1H), 6.20 (dt, J = 3.5, 2.7 Hz, 2H), 6.10-6.07 (ddt, m, 2H). **^{13}C NMR (101 MHz, Chloroform-d)** δ 139.4, 128.4, 117.0, 108.9, 108.9, 107.9, 45.0; **MS** (**MALDI-tof) m/z:** [M-H]$^+$ Calcd for $C_{15}H_{10}Br_3N_2O$ 472.83; Found 472.62 (cluster).

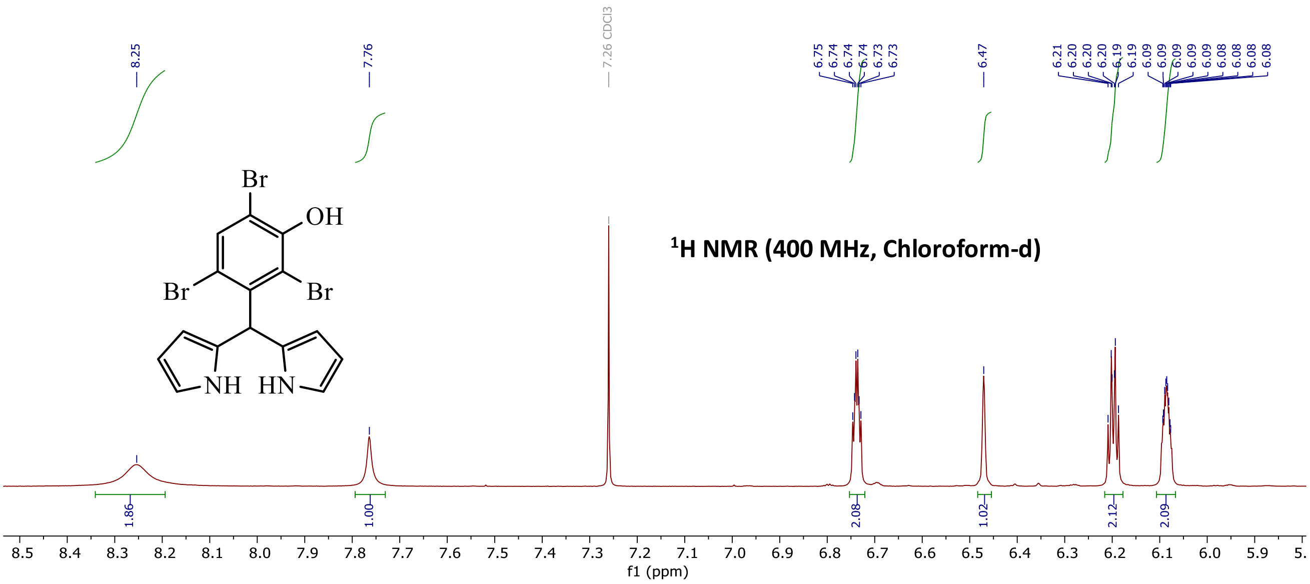

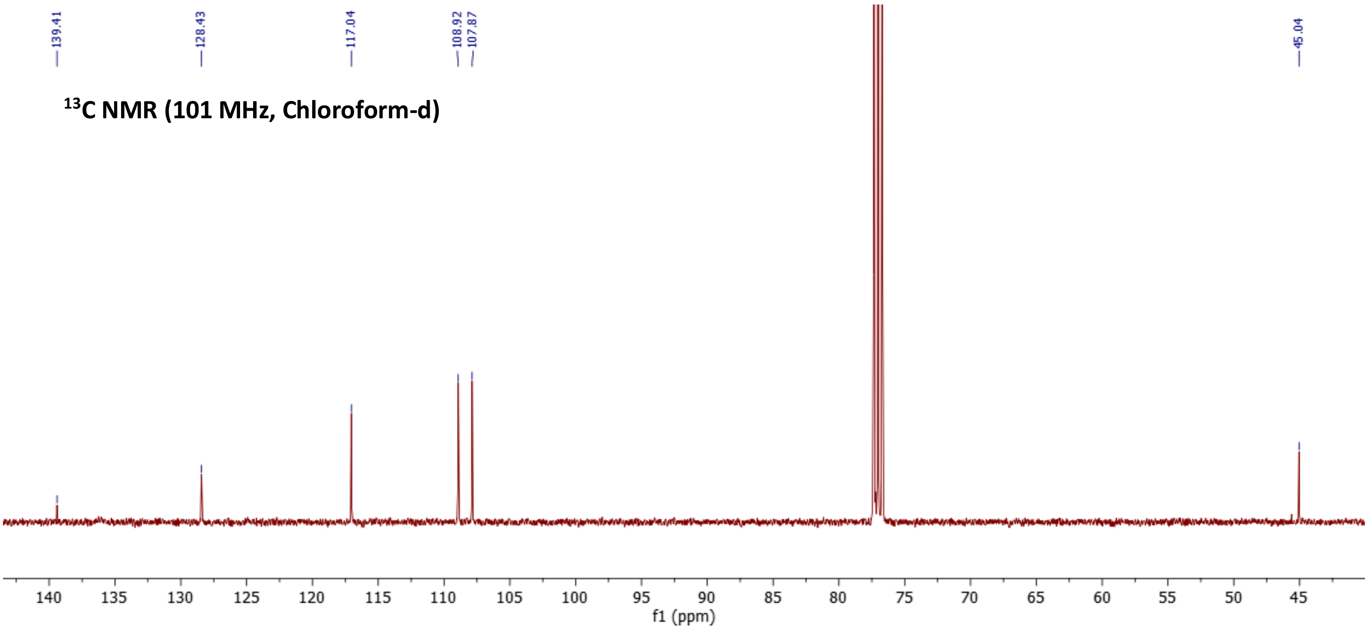

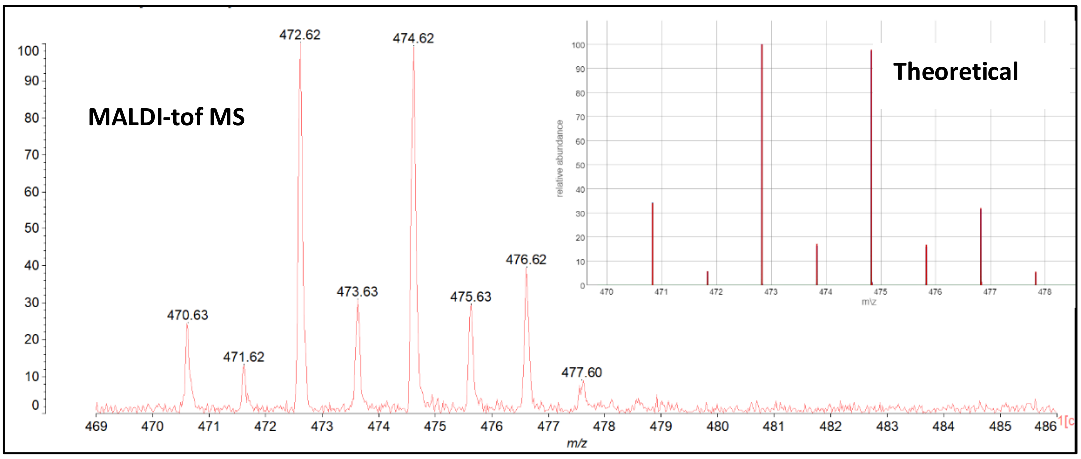

5,15-Bis-(2,4,6-tribromo-5-hydroxyphenyl)-10,20-bis-(4-methoxyphenyl)porphyrin (7)

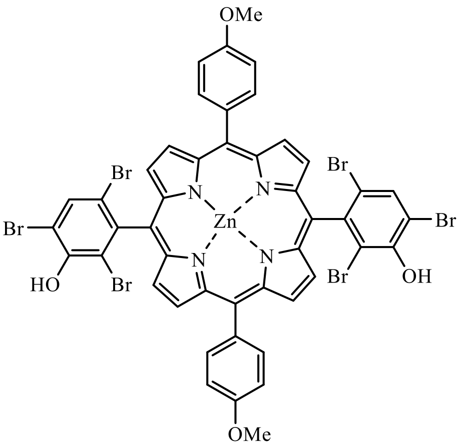

(2,4,6-Tribromo-5-hydroxyphenyl)dipyrromethane **6** (0.8g, 1.69 mmol) and 4-methoxybenzaldehyde (0.22 g, 1.69 mmol) were dissolved in anhydrous DCM (200 ml) and the reaction mixture was stirred under nitrogen. TFA (0.3 ml, 4 mmol) was added slowly at 0 ^{o}C and the solution was stirred with the progress of the reaction monitored by TLC. After 1.5h, DDQ (0.38 g, 1.69 mmol) was added, and the mixture was stirred for 60 min. Then $Zn(OAc)_2$ was dissolved in MeOH (30 ml) and added to the mixture. After 12 h, TEA (1 ml) was added, and the solvent was removed *via* rotary evaporator. The resulting solid was subjected to short column chromatography using DCM/ethyl acetate (100/5) as eluent. The solvent was removed and the residue recrystallized from DCM/MeOH afforded the *trans-Zn 7* porphyrin as purple crystals (0.527 g, 50%). **Mp** >315 ^{0}C; **^{1}H NMR (500 MHz, Chloroform-*d*)** δ 8.99 (d, *J* = 4.6 Hz, 4H) H_{β}, 8.73 (d, *J* = 4.6 Hz, 4H) H_{β}, 8.20 (s, 2H) H_{mPh}, 8.14 (d, *J* = 8.1 Hz, 4H) H_{oPh}, 7.28 (d, *J* = 8.6 Hz, 4H) H_{mPh}, 6.27, 4.10 (s, 6H) H_{CH3}.; **MS (MALDI-tof) m/z:** [M^+] Calcd for $C_{46}H_{26}Br_6N_4O_4Zn$ 1243.54; Found 1243.30 (cluster). **UV-vis, λmax (DCM)/nm** 424, ε=4.5x10^5 ; 553, ε=2.4x10^4 ; 602, ε=7.6x10^3.; **IR (KBr, cm-1):** 3451, 1604, 1493, 1433, 1376, 1335, 1231, 1173, 1061, 993, 953, 853, 795, 718, 683.

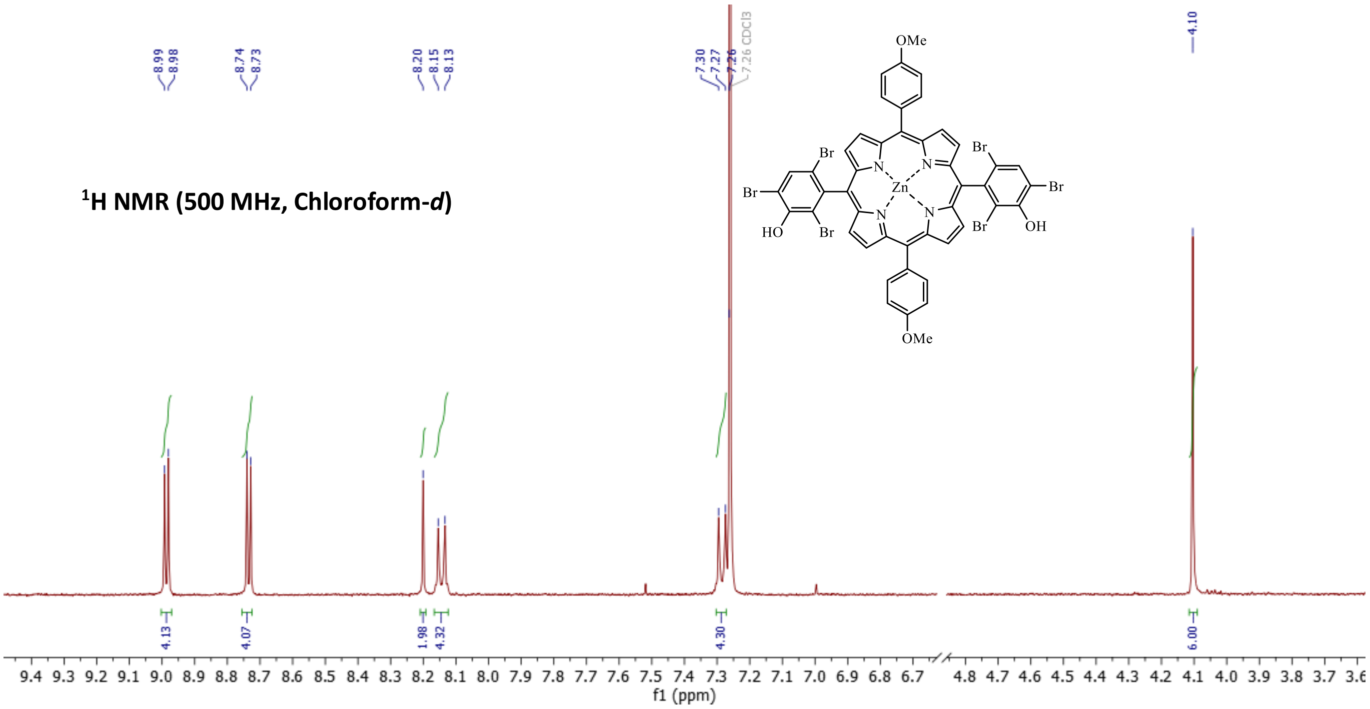

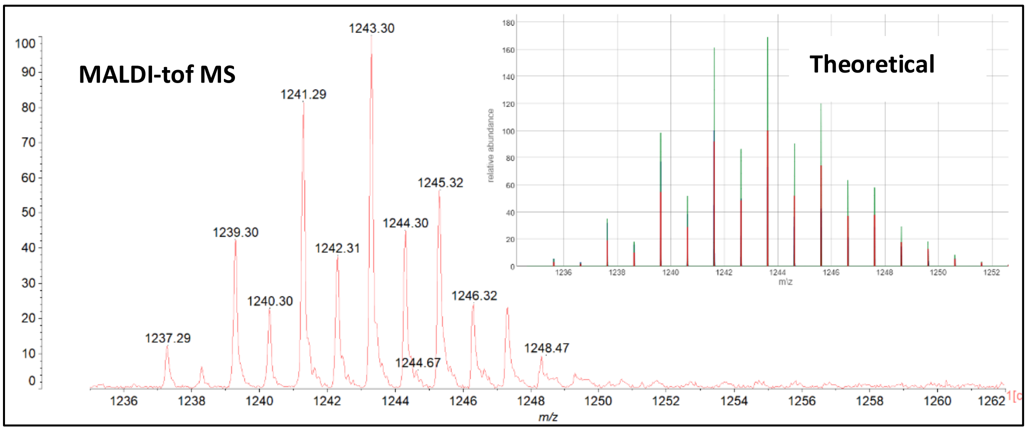

5,15 (di-hydroxyphenyl)-10,20(di-methoxyphenyl)porphyrin (4)

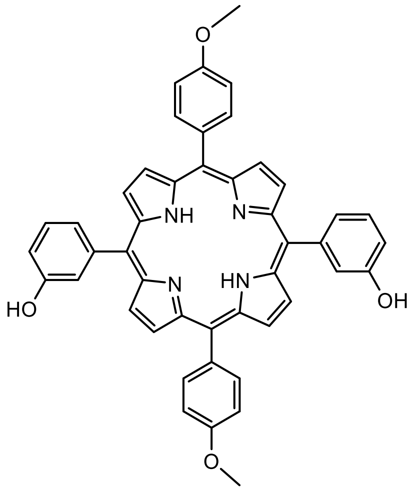

Method A

Porphyrin **7** (30mg, 0.024mmol) was dissolved in triethylsilane (6 ml) and ~5% mol% palladium dichloride (0.2 mg) was added under argon in sealed tube. The reaction was heated at 120°C for 3 days. The mixture was cooled down to room temperature and the solvent was evaporated. The resulting green solid was redissolved in DCM and treated with 2-3 drops of concentrated HCl and left stirring overnight to remove any inserted metal(s). Water was added and the mixture extracted with DCM/TEA (2 drops TEA per 100 ml DCM, x3) dried over anhydrous $MgSO_4$ and the solvent removed under reduced pressure. The crude product was subjected to column chromatography on silica gel with DCM/Ethyl acetate (100:0.5) to give a purple solid which was recrystallized from DCM/MeOH to obtain porphyrin **4** (15mg, 88%).

Method B

Porphyrin **7** (10 mg, 0.08 mmol) was dissolved in triethylsilane (6 ml) and 10% Pd/C (~1mg) was added under argon in a sealed tube. The reaction was heated at 120°C for 3 days. The mixture was cooled to room temperature then the solvent was evaporated. The resulting green solid was redissolved in DCM and treated with 2-3 drops of concentrated HCl and left stirring overnight to remove inserted metal(s). Water was added and the mixture

extracted with DCM/TEA (2 drops TEA per 100 ml DCM, x3) dried over anhydrous $MgSO_4$ and the solvent removed under reduced pressure. The crude product was subjected to column chromatography on silica gel with DCM/Ethyl acetate (100:0.5) to give a purple solid which was recrystallized from DCM/MeOH to obtain porphyrin **4** (4.5mg, 79 %). **Mp** >315^{0}C; **^{1}H NMR (500 MHz, Methylene Chloride-*d_2*) δ** 8.90 (d, *J* = 4.3 Hz, 4H), 8.89 (d, *J* = 4.3 Hz, 4H), 8.12 (d, *J* = 8.3 Hz, 4H), 7.79 (dt, *J* = 7.5, 1.2 Hz, 2H), 7.69 (br-t, J = 1.2 Hz, 2H), 7.62 (t, J = 7.5 Hz, 2H), 7.31 (d, *J* = 8.3 Hz, 4H), 7.28 (dd, J = 7.5, 1.2 Hz, 2H), 4.01 (s, 6H), -2.86 (br-s, 2H); **^{13}C NMR (126 MHz, Methylene Chloride-*d_2*)** δ 159.7, 154.3, 143.8, 135.37, 134.4, 127.89, 127.75, 122.0, 119.6, 114.8, 112.4, 55.69 (discernable signals); **MS** (**MALDI-tof) m/z:** **[**M^+] Calcd for $C_{46}H_{34}N_4O_4$ 706.26; Found 706.99 (cluster). **UV-vis, λmax (DCM)/nm** 421, ε=4.19x10^5 ; 519, ε=1.85x10^4 ; 556, ε=8.98x10^3 ; 596, ε=5.59x10^3 ; 653, ε=2.52x10^3.

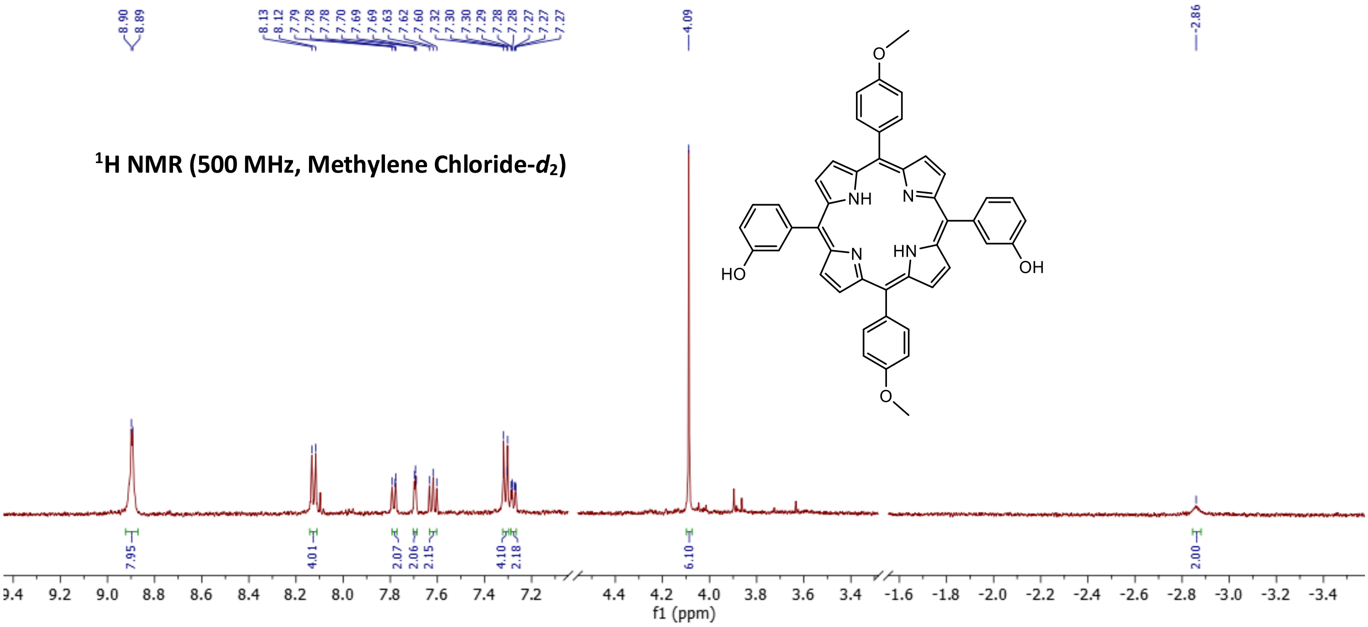

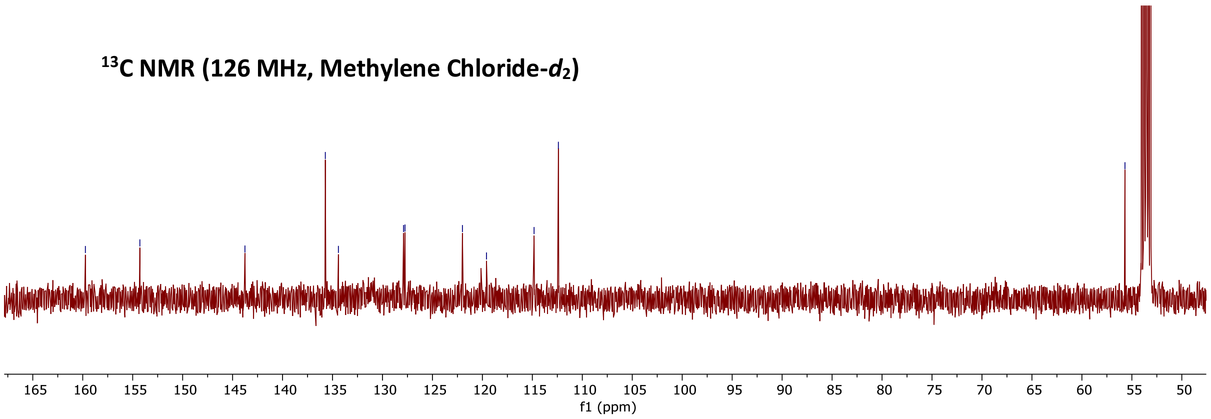

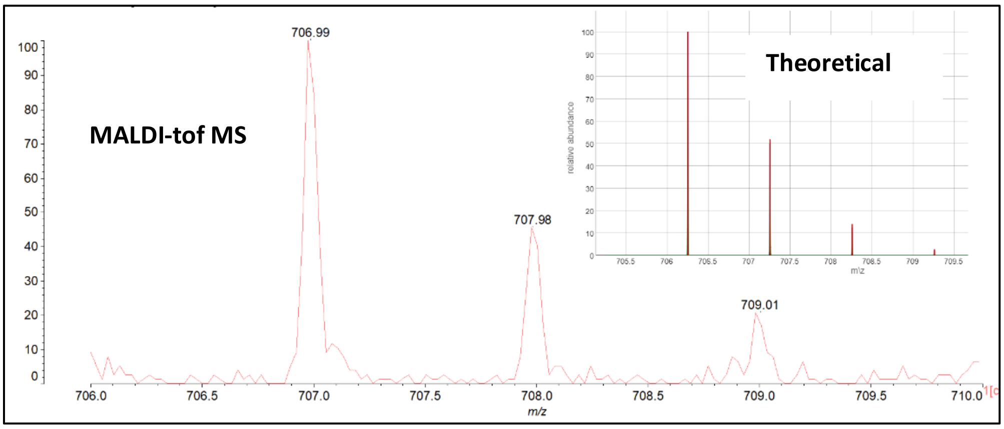

Porphyrin ditriflate (8)

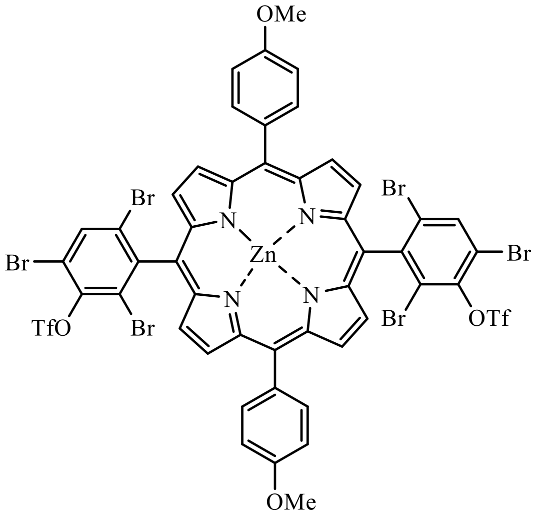

Porphyrin **7** (0.1 g, 0.080 mmol) was dissolved in anhydrous DCM (100 ml). Pyridine (0.095 g, 1.19 mmol) was then added, and the reaction mixture was stirred under N_2. Triflic anhydride (0.334 g, 1.25 mmol) was added slowly at 0°C, and the solution was stirred overnight. The mixture was extracted with water and $NaHCO_3$, and then dried over $MgSO_4$. After the solvent was removed, the resultant solid was redissolved in a mixture of DCM/MeOH in the presence of Zn $(OAc)_2$ to convert the free porphyrin to Zn porphyrin. The reaction was complete after stirring at room temperature for 2.5 hours. The solvent was evaporated, and the mixture purified by column chromatography using 100% DCM to collect the pure porphyrin **8** as a purple amorphous solid (0.097 g, 81%). **^{1}H NMR (400 MHz, Chloroform-*d*)** δ 9.02 (d, J = 4.7 Hz, 4H), 8.69 (d, J = 4.7 Hz, 4H), 8.39 (2 x s, 2H), 8.15 – 8.12 (m, 4H) H_{oPh}, 7.29 (d, J = 8.6 Hz, 4H), 4.11 (s, 6H); **^{13}C NMR (101 MHz, $CDCl_3$)** δ 159.5, 151.3, 148.3, 146.1, 135.8, 135.5, 134.4, 133.8, 129.6, 127.8, 124.59, 124.3, 121.7, 117.9, 112.2, 55.6 (discernable signals). **UV-vis, λmax (DCM)/nm** 427, ε=5.6x10^5 ; 554, ε=4.6 x10^4 ; 593, ε=2.7x10^4.

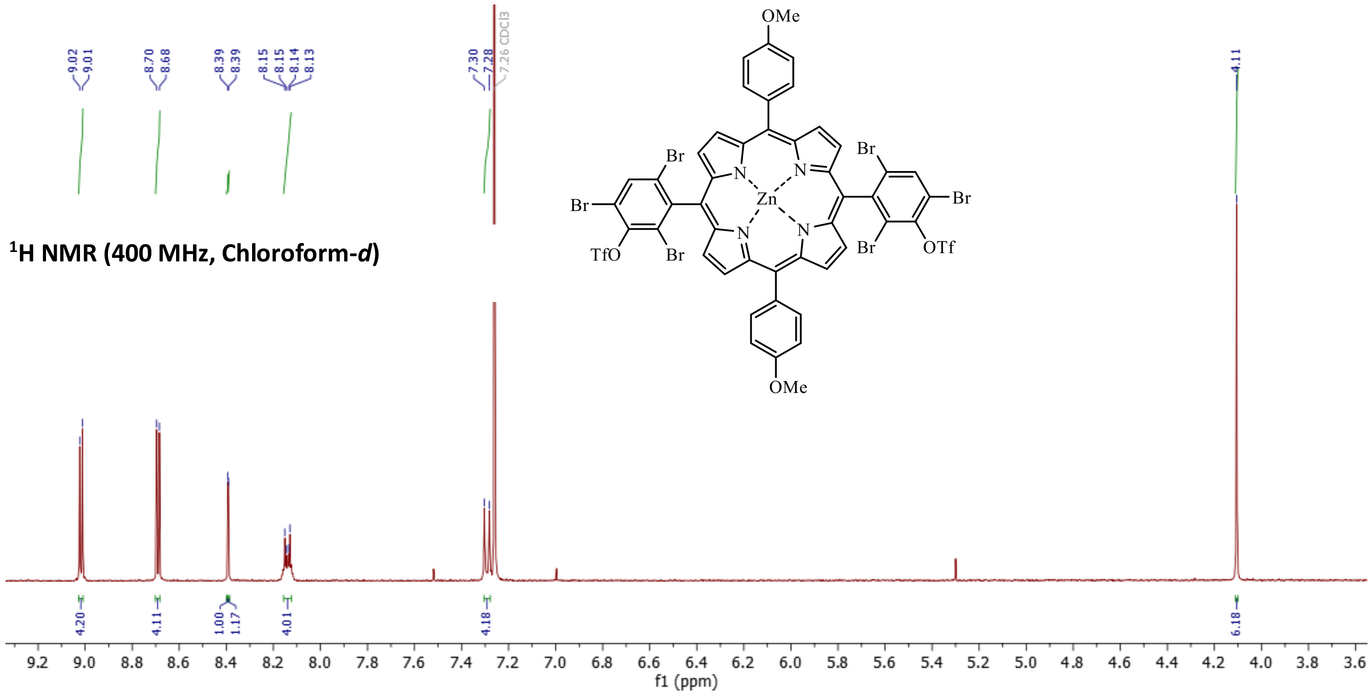

1H NMR (400 MHz, Chloroform-d)
9.02
9.01
8.70
8.68
8.39
8.39
8.15
8.15
8.14
8.13
7.30
7.28
7.26 CDCl3
4.11
4.20
4.11
1.00
1.17
4.01
4.18
6.18
f1 (ppm)
OMe
Br
Br
TfO
Br
N
Zn
N
N
N
Br
Br
Br
OTf
OMe

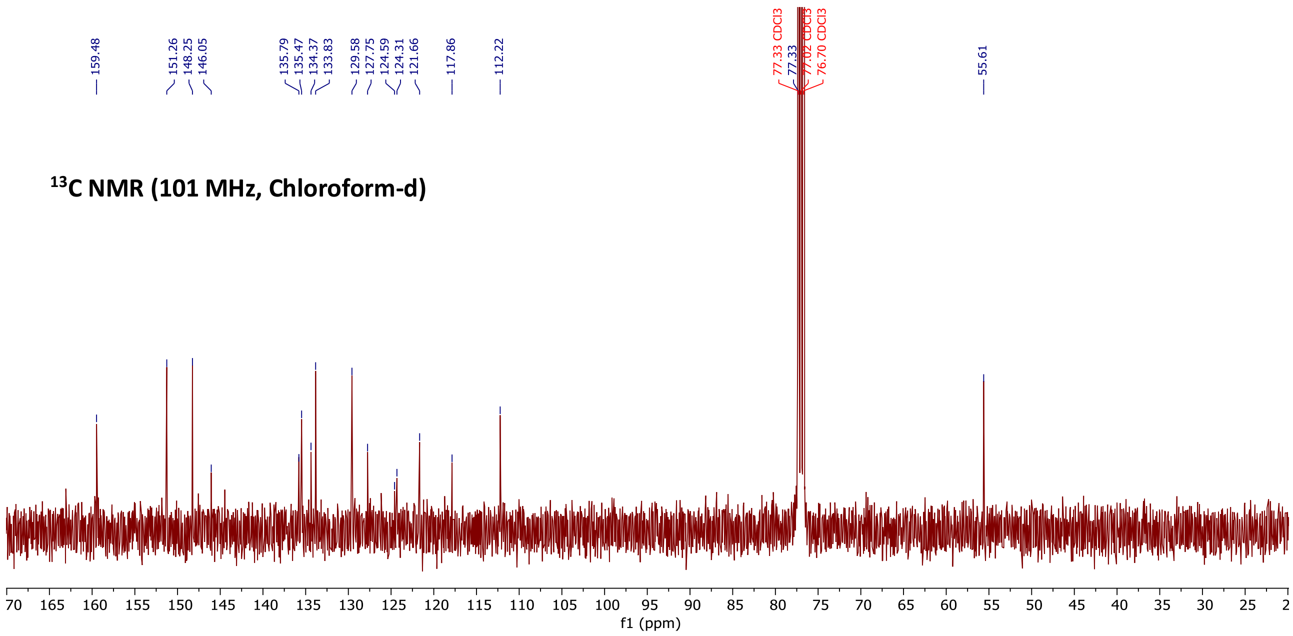

13C NMR (101 MHz, Chloroform-d)
159.48
151.26
148.25
146.05
135.79
135.47
134.37
133.83
129.58
127.75
124.59
124.31
121.66
117.86
112.22
77.33 CDCl3
77.33
77.02 CDCl3
76.70 CDCl3
55.61
f1 (ppm)

Reduction of porphyrin 8 to 5,15-diphenyl-10,20-bis-(4-methoxyphenyl)porphyrin (2)

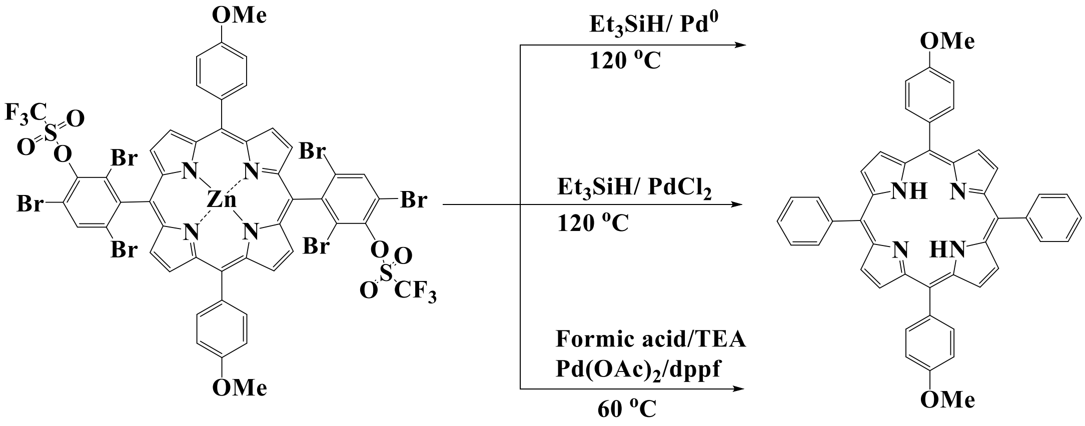

Full reduction of porphyrin **8** was attempted by following the previously used reduction conditions employed for porphyrin **7**. Separately, porphyrin **8** was dissolved in triethylsilane (as reactant and solvent) and Pd/C or $PdCl_2$ added. The reactions were heated at 120 °C and monitored periodically for a week. The reactions were broadly similar, each very slow and, in the case of Pd/C further complicated by triflate hydrolysis. Analysis of an aliquot (after HCl treatment) indicates loss of bromine is faster than triflate (~972 = loss of all 6 Br; ~823 = loss of 6 x Br and 1 x OTf) but the peak cluster ~901 corresponds to loss of 5 x Br and 1 x OTf indicating that reduction is possible. The peak cluster at 788 corresponds to loss of 5 x Br and hydrolysis of both triflates, and other peaks (e.g. ~943) indicate competing hydrolysis.

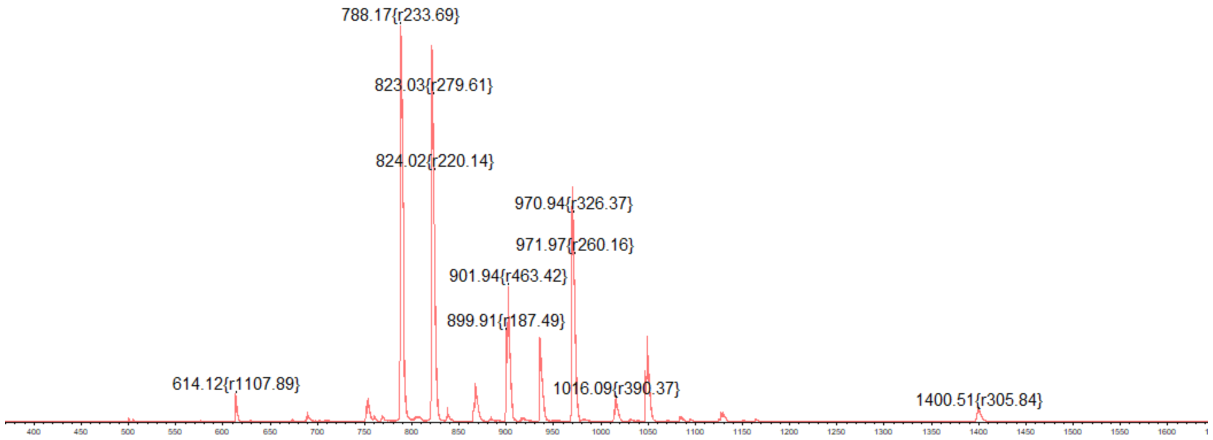

MALDI-tof MS obtained for the reduction of porphyrin **8** under Pd/C+TES conditions after 3 days.

Palladium chloride catalysed reactions are better but were still deemed too problematic to justify extended examination. For example, the reaction after 3 weeks shows minimal hydrolysis and formation of the fully reduced porphyrin **2**, but mono- and ditriflate remain.

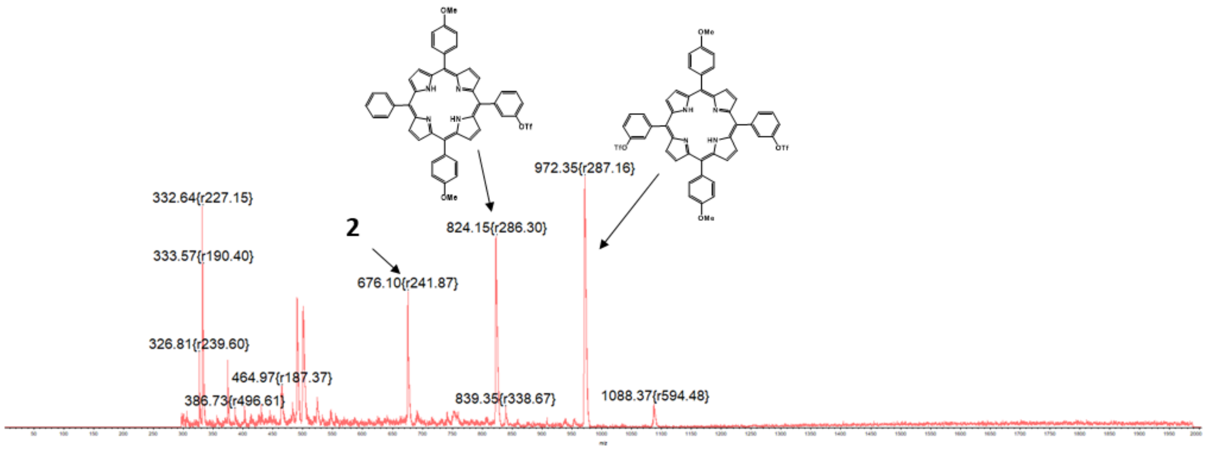

MALDI-tof MS obtained for the synthesis of porphyrin **2** under $PdCl_2$ conditions after three weeks.

Alternative conditions, using a mixture of formic acid, triethylamine (TEA), and $Pd(OAc)_2$/dppf in DMF at 60°C also indicated that reduction was possible but not practical. Reactions under these conditions showed clear evidence for significant hydrolysis competing with reduction of both triflate and bromide.

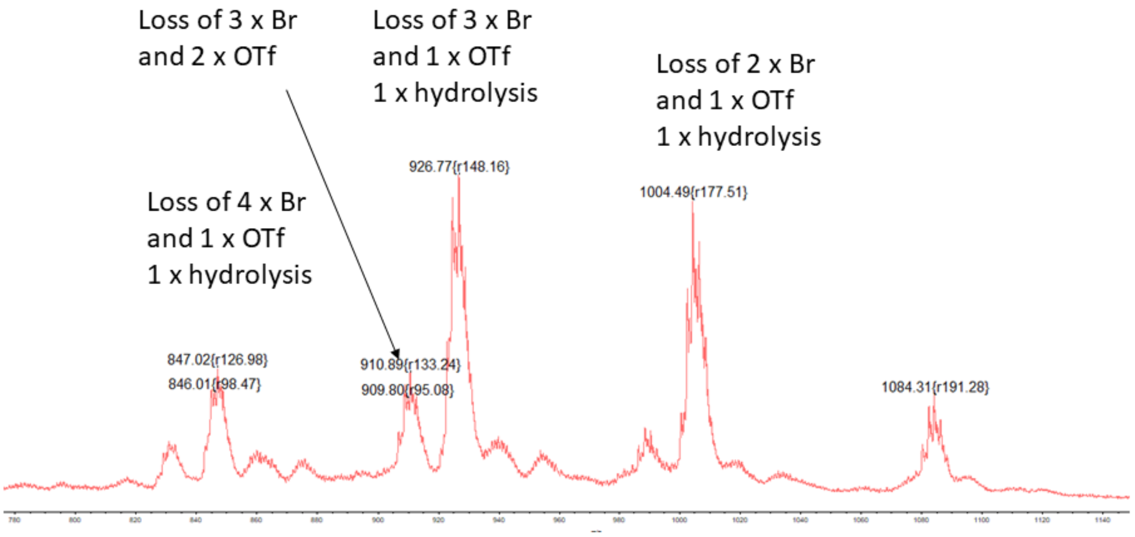

MALDI-tof MS obtained for the synthesis of porphyrin **2** under Pd-formate conditions after 7 days.

2,6- dibromophenylDPM (11)[3]

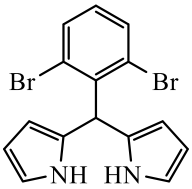

A modified version of the Lindsey method was used to synthesis the 2,6-dibromophenyldipyrromethane. To a dry round bottomed flask a mixture of 2,6-dibromobenzaldehyde (2 g, 7.57 mmol) and freshly distilled pyrrole (12.73 g, 13.16 ml, 190 mmol) were stirred at room temperature under argon for 20 min. Trifluoroacetic acid (0.06 ml, 0.79 mmol) was added slowly to the mixture and left stirring for 5 min. NaOH (0.1 M) was added to quench the reaction then it was extracted with ethyl acetate. The organic layer was washed with water (x 3) and dried over anhydrous Na_2SO_4. The solvent was removed under reduced pressure to give a brown oil which was chromatographed (silica, hexane: ethyl acetate (30:1)). Crystallization from DCM/Pet ether afforded the DPM as pure yellow crystals (1.3 g, 45%). **^{1}H NMR (400 MHz, Chloroform-d)** δ 8.30 (br-s, 2H), 7.58 (d, J = 8.0 Hz, 2H), 6.97 (t, J = 8.0 Hz, 1H), 6.76-6.72 (m, 2H), 6.55 (s, 1H), 6.22-6.19 (m, 2H), 6.12-6.08 (m, 2H). **^{13}C NMR (101 MHz, Chloroform-d)** δ 139.4, 133.5 (br), 129.4, 129.2, 116.9, 108.8, 107.7, 44.6.

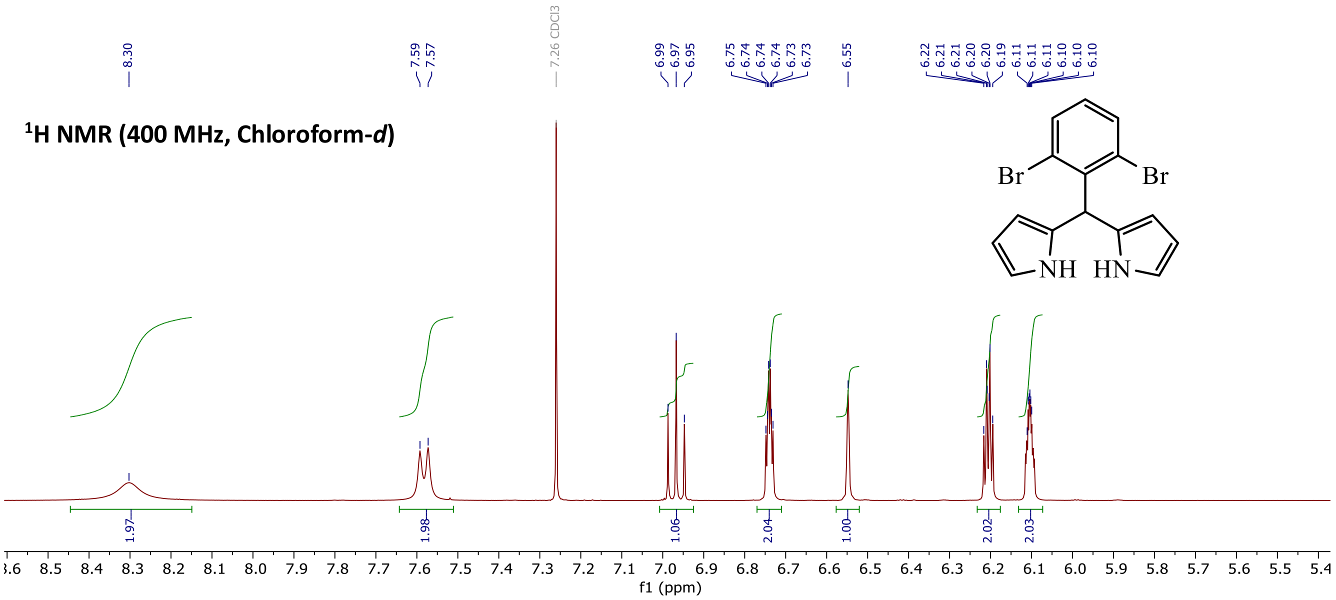

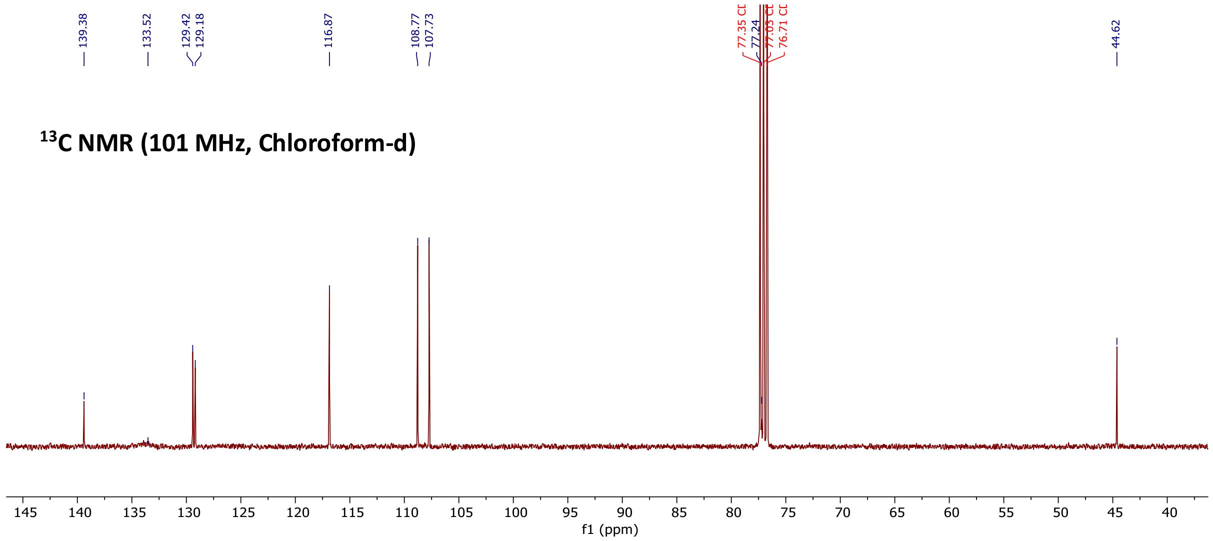

5,15-bis-(2,6-dibromophenyl)-10,20-bis-(4-methoxyphenyl)porphyrin (12)

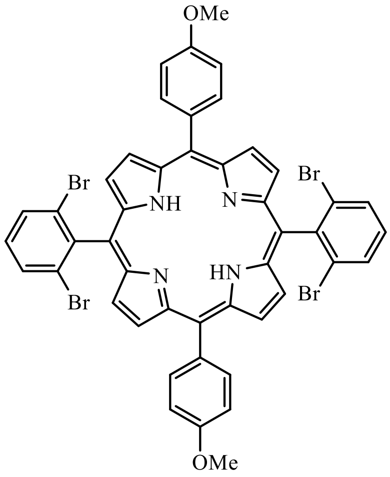

2,6-Dibromophenyldipyrromethane **11** (0.381g, 1 mmol) and 4-methoxybenzaldehyde (0.136 g, 1 mmol) were dissolved in anhydrous DCM (100 ml) and the reaction mixture was stirred under inert atmosphere for 20 minutes. TFA (0.15 ml, 2 mmol) was added slowly to the mixture, at 0 °C, and the mixture was stirred with the progress of the reaction monitored by TLC. After 1.5h, DDQ (0.22 g, 1 mmol) was added, and the mixture was stirred for 60 min. TEA (1 ml) was added to neutralize. The reaction mixture was eluted through a short silica column with DCM and washed with DCM. Crystallization from DCM/hexane afforded the *trans-* porphyrin as pure purple crystals (0.28 g, 57 %). **Mp** >315^{0}C ; **^{1}H NMR (400 MHz, Chloroform-*d*)** δ 8.88 (d, *J* = 4.8 Hz, 4H), 8.64 (d, *J* = 4.8 Hz, 4H), 8.15 (d, *J* = 8.5 Hz, 4H), 8.03 (d, *J* = 8.1 Hz, 4H), 7.54 (t, *J* = 8.1 Hz, 2H), 7.28 (d, *J* = 8.5 Hz, 4H), 4.02 (s, 6H), -2.53 (s, 2H); **MS (MALDI-tof) m/z:** $[M+H]^+$ Calcd for $C_{46}H_{31}Br_6N_4O_2$ 990.91; Found 990.62 (cluster). **UV-vis, λmax (DCM)/nm (A):** 422 (0.79), 519 (0.036) ,556 (0.012) ,596 (0.011), 652 (0.007).

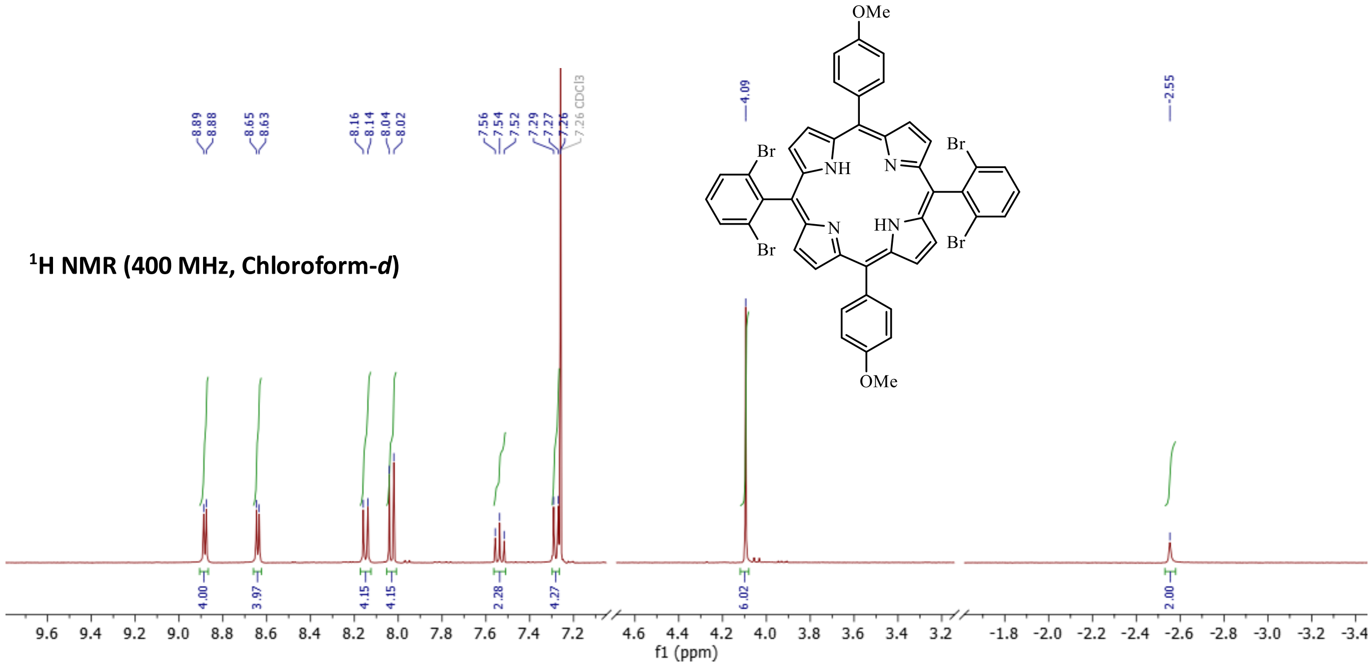

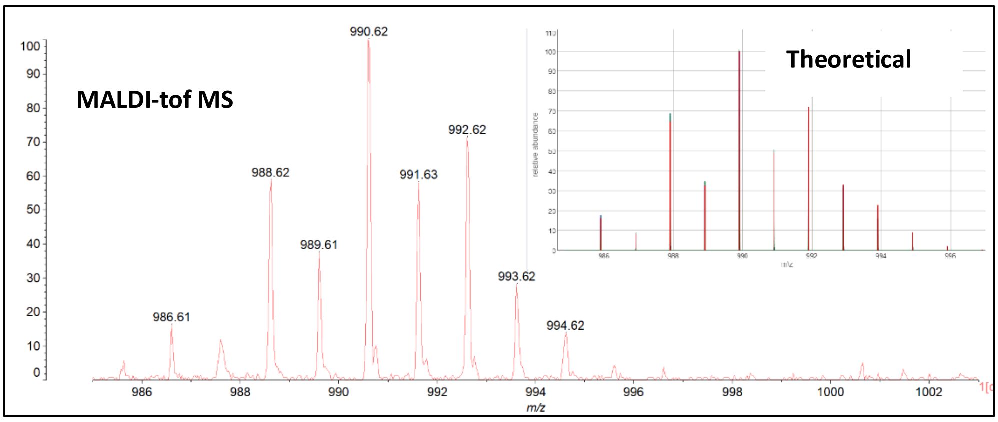

5,15-Bis-(2,6-dibromophenyl)-10,20-bis-(4-methoxyphenyl)porphyrinato zinc (13)

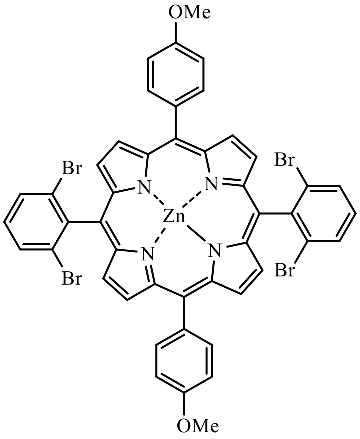

Porphyrin **12** (0.2 g, 0.20 mmol) was dissolved in DCM (150 ml). $Zn(OAc)_2$ (0.07g, 0.3 mmol) in MeOH (15 ml) was added and the mixture heated at reflux overnight. The solvent was evaporated and the resulting purple solid was redissolved in THF and passed through a silica pad eluting with THF. The solvent was removed and crystallization from THF/MeOH afforded the *trans-Zn* porphyrin **13** as purple crystals (0.198 g, 94 %). The crystals were of sufficient size and quality for X-ray crystallography. **Mp** >315^{0}C ; **^{1}H NMR** (400 MHz, Chloroform-*d*) δ 8.98 (d, *J* = 4.6 Hz, 4H), 8.73 (d, *J* = 4.6 Hz, 4H), 8.16 (d, *J* = 8.5 Hz, 4H), 8.03 (d, *J* = 8.1 Hz, 4H), 7.53 (t, *J* = 8.1 Hz, 2H), 7.27 (d, *J* = 8.5 Hz, 4H),

4.09 (s, 6H); **MS (MALDI-tof) m/z:** $[M+H]^+$ Calcd for $C_{46}H_{28}Br_6N_4O_2Zn$ 1047.82; Found 1047.69 (cluster). **UV-vis, λmax (DCM)/nm (A):** 424 (0.95), 552 (0.038), 594 (0.005).

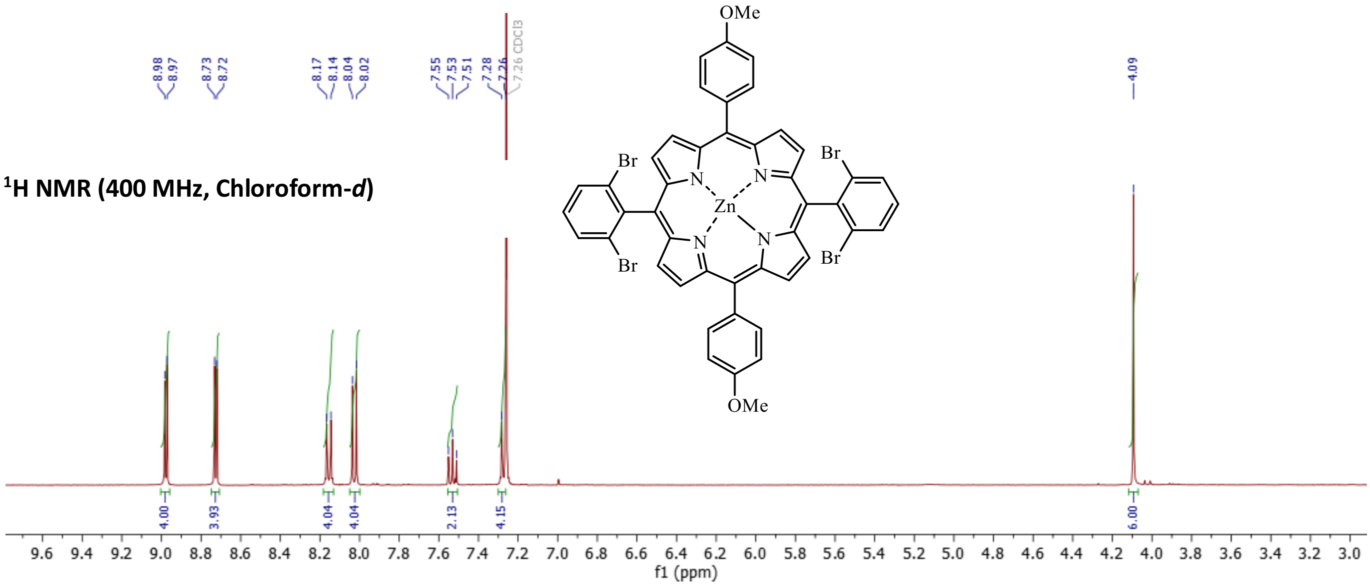

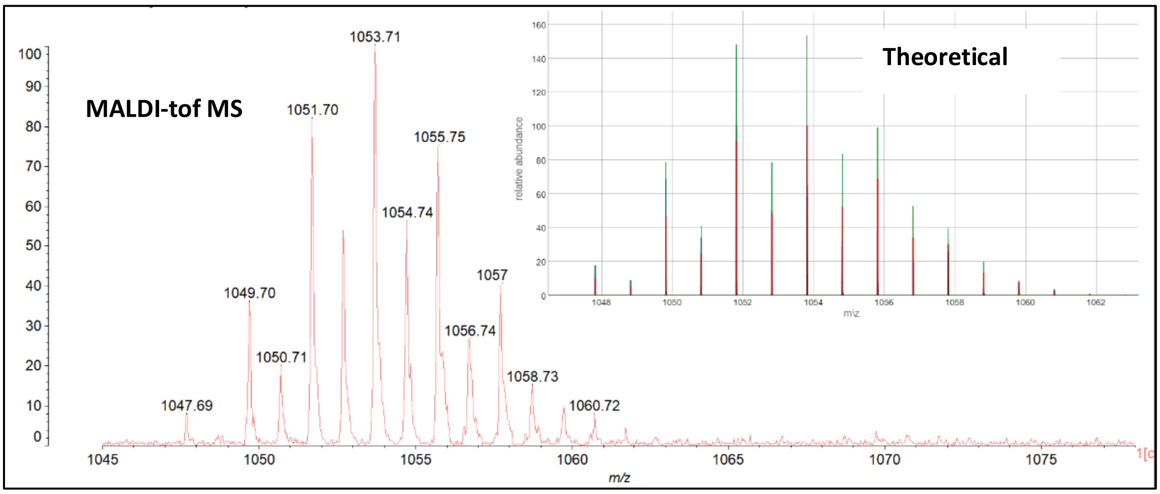

5,15-Diphenyl-10,20-bis-(4-methoxyphenyl)porphyrin (2)[4]

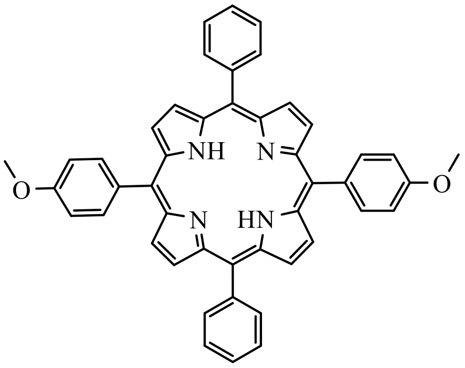

Method A

Porphyrin **12** (30mg, 0.028mmol), a 1.1 mixture of THF and triethyl silane (6 ml) and 10% Pd/C (~1 mg) was added into a sealed tube. The reaction was heated at 120 °C for 3days. After cooling, the solvent was evaporated and the

resulting solid was redissolved in DCM, treated with 2-3 drops of TEA and the solvent removed under reduced pressure. The crude product which was subjected to column chromatography on silica gel with DCM/hexane (1:1) to give a purple solid which was recrystallized from DCM/hexane to obtain porphyrin **4** (12mg, 60%). ^{1}H NMR (400 MHz, Chloroform-*d*) δ 8.87 (d, *J* = 4.8 Hz, 4H), 8.83 (d, *J* = 4.8 Hz, 4H), 8.22 (dd, *J* = 7.7, 1.6 Hz, 4H), 8.13 (d, *J* = 8.6 Hz, 4H), 7.83 – 7.70 (m, 6H), 7.29 (d, *J* = 8.6 Hz, 4H), 4.10 (s, 6H), -2.76 (s, 2H).

Method B

Porphyrin **13** (30mg, 0.028mmol), a 1:1 mixture of THF and triethyl silane (6 ml) and palladium dichloride (~0.2 mg) were added into a sealed tube. The reaction was heated at 120 ^{o}C for 3 days, cooled down and the solvent evaporated. The resulting green solid was redissolved in DCM and treated with 2-3 drops of concentrated HCl and left stirring overnight to remove any Pd and Zn that was inserted into the porphyrin. The mixture neutralised by adding further DCM+TEA, extracted with water (x3) and dried ($MgSO_4$). The solvent was removed under reduced pressure to give a crude product which was subjected to column chromatography on silica gel with DCM/hexane (1:1) to give a purple solid which was recrystallized from DCM/hexane to obtain compound **4** (14mg, 74%).

References

1. Littler, B. J.; Miller, M. A.; Hung, C-H.; Wagner, R. W.; O'Shea, D. F.; Boyle, P. D.; Lindsey, J. S. Refined synthesis of 5-substituted dipyrromethanes. *J. Org. Chem.*, **1999**, *64*, 1391-1396.

2. D'Souza, F.; Deviprasad, G. R.; El-Khouly, M. E.; Fujitsuka, M.; Ito, O. Probing the donor-acceptor proximity on the physicochemical properties of porphyrin-fullerene dyads: "Tail-on" and "Tail-off" binding approach. *J. Am. Chem. Soc.,* **2001**, *123*, 5277-5284.

3. Lu, H.; Li, C.; Jiang, H.; Lizardi, C. L.; Zhang, X. P. Chemoselective amination of propargylic C(sp^3)-H bonds by cobalt(II)-based metalloradical catalysis. *Angew. Chem.Int. Ed.,* **2014**, *53*, 7028-7032.

4. Shi, B.; Boyle, R. W. Synthesis of unsymmetrically substituted *meso*-phenylporphyrins by Suzuki cross coupling reactions. *J. Chem. Soc. Perkin Trans. 1.* **2002**, 1397-1400.

Crystal data for porphyrin 13 (atom numbering on p31)

Crystal data and structure refinement for
[Zn (OH_2) Porph-$(C_6H_3-Br_2)_2$,$(C_6H_4-OMe)_2$],2$(CHCl_3)$

Identification code	isabf1450
Elemental formula	C46 H30 Br4 N4 O3 Zn, 2(C H Cl3)
Formula weight	1310.48
Crystal system, space group	Monoclinic, C2/c (no. 15)
Unit cell dimensions	a = 20.5852(3) Å α = 90 °
	b = 9.21383(14) Å β = 109.102(2) °
	c = 26.0515(4) Å γ = 90 °
Volume	4669.06(12) $Å^3$
Z, Calculated density	4, 1.864 Mg/m^3
F(000)	2576
Absorption coefficient	8.299 mm^{-1}
Temperature	99.98(11) K
Wavelength	1.54184 Å
Crystal colour, shape	purple prism
Crystal size	0.24 x 0.20 x 0.15 mm
Crystal mounting:	on a small loop, in oil, fixed in cold N_2 stream
On the diffractometer:	
Theta range for data collection	8.065 to 72.469 °
Limiting indices	-24<=h<=25, -11<=k<=8, -31<=l<=31
Completeness to theta = 67.684	99.2 %
Absorption correction	Semi-empirical from equivalents
Max. and min. transmission	1.00000 and 0.40400
Reflections collected (not including absences)	16767
No. of unique reflections	4403 [R(int) for equivalents = 0.044]
No. of 'observed' reflections ($I > 2\sigma_I$)	4021
Structure determined by:	dual methods, in SHELXT
Refinement:	Full-matrix least-squares on F^2, in SHELXL
Data / restraints / parameters	4403 / 0 / 303
Goodness-of-fit on F^2	1.048

Final R indices ('observed' data)	R_1 = 0.043, wR_2 = 0.112
Final R indices (all data)	R_1 = 0.047, wR_2 = 0.114

Reflections weighted:
$w = [\sigma^2(Fo^2)+(0.0622P)^2+23.1867P]^{-1}$ where $P=(Fo^2+2Fc^2)/3$

Extinction coefficient	n/a
Largest diff. peak and hole	1.58 and -1.13 e.Å^{-3}
Location of largest difference peak	near Cl(24)

Table 1. Atomic coordinates (x 10^5) and equivalent isotropic displacement parameters ($Å^2$ x 10^4). U(eq) is defined as one third of the trace of the orthogonalized Uij tensor. E.s.ds are in parentheses.

	x	y	z	U(eq)
O(1)	50000	21230(50)	75000	254(8)
Zn	50000	44608(8)	75000	171(2)
N(1)	60275(14)	47780(40)	79254(11)	186(6)
C(2)	63222(17)	47210(40)	84808(13)	182(7)
C(3)	70594(17)	47250(40)	86241(14)	207(7)
C(4)	72070(17)	47890(40)	81543(14)	210(7)
C(5)	65623(18)	48240(40)	77145(14)	201(7)
C(6)	64871(17)	48220(40)	71587(14)	194(7)
N(7)	52214(15)	47670(40)	67889(11)	195(6)
C(8)	58631(18)	47770(40)	67347(14)	196(7)
C(9)	57938(19)	47140(50)	61646(14)	248(8)
C(10)	51164(18)	46540(40)	58849(14)	229(8)
C(11)	47570(18)	46880(40)	62761(14)	195(7)
C(12)	59607(18)	46490(40)	88518(13)	185(7)
C(13)	63642(17)	43920(50)	94373(14)	212(8)
C(14)	64629(18)	29820(40)	96468(14)	212(7)
Br(14)	61463(2)	14050(5)	91575(2)	263.5(13)
C(15)	67930(19)	26780(50)	101881(16)	269(8)
C(16)	70501(19)	38070(50)	105440(15)	281(9)
C(17)	69820(19)	52200(50)	103619(15)	274(9)
C(18)	66371(18)	54910(50)	98141(15)	244(8)
Br(18)	65431(2)	74595(5)	95811(2)	306.8(14)
C(61)	71457(17)	48360(40)	70270(13)	209(8)
C(62)	73898(19)	35940(50)	68519(15)	235(8)
C(63)	80479(19)	35290(40)	68049(15)	230(8)
C(64)	84570(18)	47570(40)	69308(14)	225(8)
C(65)	82068(18)	60480(40)	70656(15)	231(8)
C(66)	75555(18)	60740(50)	71170(14)	231(8)
O(64)	91291(13)	47950(30)	69305(11)	257(6)
C(641)	94380(20)	34410(50)	68636(17)	280(8)
C(21)	55560(20)	1080(60)	59412(19)	379(10)
Cl(22)	58260(6)	8428(15)	65984(4)	436(3)
Cl(23)	60132(8)	8805(15)	55459(6)	549(4)
Cl(24)	56338(10)	-17812(17)	59616(8)	707(5)

Table 2. Molecular dimensions. Bond lengths are in Ångstroms, angles in degrees. E.s.ds are in parentheses.

O(1)-Zn	2.154(4)	Zn-N(7)	2.067(3)
Zn-N(1)	2.062(3)		
N(1)-Zn-N(1)#1	163.71(19)	N(7)#1-Zn-N(7)	164.33(19)
N(1)-Zn-N(7)#1	89.27(11)	N(1)-Zn-O(1)	98.15(9)
N(1)-Zn-N(7)	88.52(11)	N(7)-Zn-O(1)	97.83(9)
O(1)-H(1O)	0.81(6)	C(14)-C(15)	1.379(5)
N(1)-C(2)	1.375(4)	C(14)-Br(14)	1.902(4)
N(1)-C(5)	1.383(4)	C(15)-C(16)	1.379(6)
C(2)-C(12)	1.401(5)	C(16)-C(17)	1.377(7)
C(2)-C(3)	1.439(5)	C(17)-C(18)	1.393(5)
C(3)-C(4)	1.355(5)	C(18)-Br(18)	1.903(4)
C(4)-C(5)	1.442(5)	C(61)-C(62)	1.386(6)
C(5)-C(6)	1.405(5)	C(61)-C(66)	1.392(6)
C(6)-C(8)	1.394(5)	C(62)-C(63)	1.401(5)
C(6)-C(61)	1.504(5)	C(63)-C(64)	1.385(6)
N(7)-C(11)	1.367(4)	C(64)-O(64)	1.384(4)
N(7)-C(8)	1.374(4)	C(64)-C(65)	1.387(6)
C(8)-C(9)	1.446(5)	C(65)-C(66)	1.390(5)
C(9)-C(10)	1.347(5)	O(64)-C(641)	1.437(5)
C(10)-C(11)	1.443(5)		
C(11)-C(12)#1	1.405(5)	C(21)-Cl(24)	1.747(5)
C(12)-C(13)	1.498(5)	C(21)-Cl(22)	1.754(5)
C(13)-C(18)	1.394(6)	C(21)-Cl(23)	1.758(5)
C(13)-C(14)	1.398(6)		
Zn-O(1)-H(1O)	129(4)	C(11)#1-C(12)-C(13)	115.8(3)
H(1O)-O(1)-H(1O)#1	101(8)	C(18)-C(13)-C(14)	115.2(3)
C(2)-N(1)-C(5)	106.6(3)	C(18)-C(13)-C(12)	124.3(4)
C(2)-N(1)-Zn	125.6(2)	C(14)-C(13)-C(12)	120.4(3)
C(5)-N(1)-Zn	126.9(2)	C(15)-C(14)-C(13)	123.2(4)
N(1)-C(2)-C(12)	125.2(3)	C(15)-C(14)-Br(14)	118.5(3)
N(1)-C(2)-C(3)	109.7(3)	C(13)-C(14)-Br(14)	118.3(3)
C(12)-C(2)-C(3)	125.0(3)	C(16)-C(15)-C(14)	119.1(4)
C(4)-C(3)-C(2)	107.2(3)	C(17)-C(16)-C(15)	120.6(4)
C(3)-C(4)-C(5)	107.4(3)	C(16)-C(17)-C(18)	118.8(4)
N(1)-C(5)-C(6)	125.2(3)	C(17)-C(18)-C(13)	123.0(4)
N(1)-C(5)-C(4)	109.2(3)	C(17)-C(18)-Br(18)	117.5(3)
C(6)-C(5)-C(4)	125.5(3)	C(13)-C(18)-Br(18)	119.5(3)
C(8)-C(6)-C(5)	125.4(3)	C(62)-C(61)-C(66)	117.8(3)
C(8)-C(6)-C(61)	119.0(3)	C(62)-C(61)-C(6)	121.3(3)
C(5)-C(6)-C(61)	115.6(3)	C(66)-C(61)-C(6)	120.7(3)
C(11)-N(7)-C(8)	106.8(3)	C(61)-C(62)-C(63)	122.0(4)
C(11)-N(7)-Zn	125.5(2)	C(64)-C(63)-C(62)	118.4(4)
C(8)-N(7)-Zn	126.5(2)	O(64)-C(64)-C(63)	123.4(4)
N(7)-C(8)-C(6)	125.9(3)	O(64)-C(64)-C(65)	115.6(3)
N(7)-C(8)-C(9)	109.3(3)	C(63)-C(64)-C(65)	120.9(3)
C(6)-C(8)-C(9)	124.8(3)	C(64)-C(65)-C(66)	119.2(4)
C(10)-C(9)-C(8)	107.1(3)	C(65)-C(66)-C(61)	121.4(4)
C(9)-C(10)-C(11)	107.2(3)	C(64)-O(64)-C(641)	117.4(3)
N(7)-C(11)-C(12)#1	125.3(3)		
N(7)-C(11)-C(10)	109.6(3)	Cl(24)-C(21)-Cl(22)	111.0(3)
C(12)#1-C(11)-C(10)	125.1(3)	Cl(24)-C(21)-Cl(23)	111.1(2)
C(2)-C(12)-C(11)#1	126.2(3)	Cl(22)-C(21)-Cl(23)	110.9(3)
C(2)-C(12)-C(13)	117.9(3)		

Symmetry transformation used to generate equivalent atoms:
#1 : 1-x, y, 1½-z

Table 3. Anisotropic displacement parameters ($Å^2 \times 10^4$) for the expression:

$$\exp\{-2\pi(h^2a^{*2}U_{11} + ... + 2hka^*b^*U_{12})\}$$

E.s.ds are in parentheses.

	U_{11}	U_{22}	U_{33}	U_{23}	U_{13}	U_{12}
O(1)	208(19)	280(20)	240(20)	0	22(16)	0
Zn	126(3)	282(4)	107(3)	0	39(2)	0
N(1)	122(13)	311(17)	131(13)	-12(12)	50(11)	-29(12)
C(2)	161(16)	258(19)	119(15)	-11(13)	34(12)	-2(14)
C(3)	148(16)	300(20)	157(16)	4(14)	25(13)	-3(14)
C(4)	137(16)	310(20)	175(17)	-12(15)	42(13)	-30(14)
C(5)	152(16)	290(20)	160(16)	3(14)	56(13)	-23(14)
C(6)	155(16)	262(19)	188(16)	-19(14)	87(13)	-18(14)
N(7)	153(13)	312(17)	114(13)	13(12)	38(11)	0(12)
C(8)	179(16)	300(20)	131(16)	29(14)	76(13)	-9(14)
C(9)	218(18)	400(20)	148(17)	-2(16)	93(14)	-37(16)
C(10)	189(17)	360(20)	132(16)	2(15)	43(13)	-13(15)
C(11)	183(16)	280(20)	122(15)	2(14)	53(13)	-17(14)
C(12)	176(16)	253(19)	121(16)	-15(13)	40(13)	-11(14)
C(13)	115(15)	410(20)	120(16)	10(15)	43(13)	23(15)
C(14)	173(16)	300(20)	174(16)	22(15)	73(13)	4(15)
Br(14)	233(2)	312(2)	239(2)	3(2)	66(2)	-4(2)
C(15)	194(17)	420(20)	202(18)	82(17)	84(14)	39(17)
C(16)	167(17)	520(30)	151(17)	52(17)	47(13)	17(17)
C(17)	172(16)	500(30)	147(17)	-59(17)	52(13)	-6(17)
C(18)	168(16)	390(20)	167(17)	-18(16)	51(13)	-19(16)
Br(18)	330(2)	336(3)	222(2)	-38(2)	47(2)	-25(2)
C(61)	149(16)	390(20)	98(15)	15(15)	49(12)	-38(15)
C(62)	193(17)	340(20)	179(17)	-11(15)	66(13)	-27(15)
C(63)	196(17)	290(20)	204(17)	-15(15)	71(14)	-15(15)
C(64)	165(16)	340(20)	173(16)	25(15)	68(13)	-7(15)
C(65)	185(17)	320(20)	196(17)	7(16)	76(13)	-58(16)
C(66)	170(16)	360(20)	161(16)	1(15)	48(13)	-31(16)
O(64)	165(12)	352(16)	272(13)	21(12)	94(10)	-3(11)
C(641)	203(18)	320(20)	330(20)	-35(17)	105(16)	8(16)
C(21)	300(20)	520(30)	350(20)	-50(20)	150(18)	40(20)
Cl(22)	426(6)	581(7)	335(5)	-89(5)	172(5)	-170(5)
Cl(23)	789(9)	504(7)	541(7)	111(6)	475(7)	138(7)
Cl(24)	1005(12)	486(8)	942(11)	-135(8)	744(10)	-133(8)

Table 4. Hydrogen coordinates (x 10^4) and isotropic displacement parameters ($Å^2$ x 10^3). The hydrogen atoms of the water molecule were located in a difference map and were refined freely. The remaining hydrogen atoms were included in idealised positions with U(iso)'s set at 1.2*U(eq) or, for the methyl group hydrogen atoms, 1.5*U(eq) of the parent carbon atoms.

	x	y	z	U(iso)
H(3)	7382	4689	8981	25
H(4)	7653	4808	8121	25
H(9)	6158	4716	6015	30
H(10)	4913	4599	5501	27
H(15)	6843	1703	10314	32
H(16)	7276	3608	10918	34
H(17)	7167	5997	10606	33
H(62)	7102	2762	6761	28
H(63)	8210	2663	6690	28
H(65)	8477	6906	7122	28
H(66)	7387	6954	7216	28
H(64A)	9911	3613	6870	42
H(64B)	9442	2781	7160	42
H(64C)	9172	3005	6515	42
H(21)	5059	352	5767	46
H(1O)	5250(30)	1560(70)	7710(20)	52(18)

Table 5. Torsion angles, in degrees. E.s.ds are in parentheses.

C(5)-N(1)-C(2)-C(12)	-179.4(4)	N(1)-C(2)-C(12)-C(13)	171.9(4)
Zn-N(1)-C(2)-C(12)	-9.9(5)	C(3)-C(2)-C(12)-C(13)	-7.1(6)
C(5)-N(1)-C(2)-C(3)	-0.2(4)	C(2)-C(12)-C(13)-C(18)	90.4(4)
Zn-N(1)-C(2)-C(3)	169.3(3)	C(11)#1-C(12)-C(13)-C(18)	-94.1(4)
N(1)-C(2)-C(3)-C(4)	0.2(4)	C(2)-C(12)-C(13)-C(14)	-92.6(4)
C(12)-C(2)-C(3)-C(4)	179.4(4)	C(11)#1-C(12)-C(13)-C(14)	82.9(4)
C(2)-C(3)-C(4)-C(5)	-0.1(4)	C(18)-C(13)-C(14)-C(15)	1.3(5)
C(2)-N(1)-C(5)-C(6)	176.8(4)	C(12)-C(13)-C(14)-C(15)	-176.0(3)
Zn-N(1)-C(5)-C(6)	7.5(6)	C(18)-C(13)-C(14)-Br(14)	-177.1(3)
C(2)-N(1)-C(5)-C(4)	0.2(4)	C(12)-C(13)-C(14)-Br(14)	5.6(4)
Zn-N(1)-C(5)-C(4)	-169.2(3)	C(13)-C(14)-C(15)-C(16)	-1.0(6)
C(3)-C(4)-C(5)-N(1)	-0.1(5)	Br(14)-C(14)-C(15)-C(16)	177.4(3)
C(3)-C(4)-C(5)-C(6)	-176.7(4)	C(14)-C(15)-C(16)-C(17)	-0.3(6)
N(1)-C(5)-C(6)-C(8)	0.3(6)	C(15)-C(16)-C(17)-C(18)	1.3(6)
C(4)-C(5)-C(6)-C(8)	176.4(4)	C(16)-C(17)-C(18)-C(13)	-1.0(5)
N(1)-C(5)-C(6)-C(61)	-178.4(4)	C(16)-C(17)-C(18)-Br(18)	179.1(3)
C(4)-C(5)-C(6)-C(61)	-2.3(6)	C(14)-C(13)-C(18)-C(17)	-0.3(5)
C(11)-N(7)-C(8)-C(6)	-178.7(4)	C(12)-C(13)-C(18)-C(17)	176.8(3)
Zn-N(7)-C(8)-C(6)	-11.2(6)	C(14)-C(13)-C(18)-Br(18)	179.6(2)
C(11)-N(7)-C(8)-C(9)	0.4(4)	C(12)-C(13)-C(18)-Br(18)	-3.3(5)
Zn-N(7)-C(8)-C(9)	168.0(3)	C(8)-C(6)-C(61)-C(62)	-74.7(5)
C(5)-C(6)-C(8)-N(7)	1.7(6)	C(5)-C(6)-C(61)-C(62)	104.0(4)
C(61)-C(6)-C(8)-N(7)	-179.7(4)	C(8)-C(6)-C(61)-C(66)	110.2(4)
C(5)-C(6)-C(8)-C(9)	-177.3(4)	C(5)-C(6)-C(61)-C(66)	-71.0(5)
C(61)-C(6)-C(8)-C(9)	1.3(6)	C(66)-C(61)-C(62)-C(63)	4.9(5)
N(7)-C(8)-C(9)-C(10)	-0.5(5)	C(6)-C(61)-C(62)-C(63)	-170.3(3)
C(6)-C(8)-C(9)-C(10)	178.7(4)	C(61)-C(62)-C(63)-C(64)	-1.0(5)
C(8)-C(9)-C(10)-C(11)	0.4(5)	C(62)-C(63)-C(64)-O(64)	176.0(3)
C(8)-N(7)-C(11)-C(12)#1	179.8(4)	C(62)-C(63)-C(64)-C(65)	-4.2(5)
Zn-N(7)-C(11)-C(12)#1	12.1(5)	O(64)-C(64)-C(65)-C(66)	-175.0(3)
C(8)-N(7)-C(11)-C(10)	-0.2(4)	C(63)-C(64)-C(65)-C(66)	5.2(5)
Zn-N(7)-C(11)-C(10)	-167.9(3)	C(64)-C(65)-C(66)-C(61)	-1.1(5)
C(9)-C(10)-C(11)-N(7)	-0.1(5)	C(62)-C(61)-C(66)-C(65)	-3.8(5)
C(9)-C(10)-C(11)-C(12)#1	179.8(4)	C(6)-C(61)-C(66)-C(65)	171.4(3)
N(1)-C(2)-C(12)-C(11)#1	-3.1(6)	C(63)-C(64)-O(64)-C(641)	-8.2(5)
C(3)-C(2)-C(12)-C(11)#1	177.9(4)	C(65)-C(64)-O(64)-C(641)	172.0(3)

Symmetry transformations used to generate equivalent atoms:
#1 : 1-x, y, 1½-z

Table 6. Hydrogen bond, in Ångstroms and degrees.

D-H...A	d(D-H)	d(H...A)	d(D...A)	<(DHA)
O(1)-H(1O)...O(64)#2	0.81(6)	2.08(6)	2.877(4)	164(6)

Symmetry transformation used to generate equivalent atoms:
#1 : 1-x, y, 1½-z #2 : 1½-x, y-½, 1½-z

Crystal structure analysis of [Zn (OH_2) Porph-(C_6H_3-Br_2)$_2$,(C_6H_4-OMe)$_2$],2($CHCl_3$)

Crystal data: $C_{46}H_{30}Br_4N_4O_3Zn$, 2($CHCl_3$), M = 1310.48. Monoclinic, space group C2/c (no. 15), a = 20.5852(3), b = 9.21383(14), c = 26.0515(4) Å, β = 109.102(2) °, V = 4669.06(12) Å^3. Z = 4, Dc = 1.864 g cm^{-3}, F(000) = 2576, T = 99.98(11) K, μ(Cu-Kα) = 83.0 cm^{-1}, λ(Cu-Kα) = 1.54184 Å.

The crystal was a purple prism. From a sample under oil, one, *ca* 0.15 x 0.20 x 0.24 mm, was mounted on a small loop and fixed in the cold nitrogen stream on a Rigaku Oxford Diffraction XtaLAB Synergy diffractometer, equipped with Cu-Kα radiation, HyPix detector and mirror monochromator. Intensity data were measured by thin-slice ω-scans. Total no. of reflections recorded, to θ_{max} = 72.5°, was 16,767 of which 4,403 were unique (Rint = 0.044); 4,021 were 'observed' with I > $2\sigma_I$.

Data were processed using the CrysAlisPro-CCD and -RED (1) programs. The structure was determined by the intrinsic phasing routines in the SHELXT program (2A) and refined by full-matrix least-squares methods, on F^2's, in SHELXL (2B). The non-hydrogen atoms were refined with anisotropic thermal parameters. The hydrogen atom on O(1) was located in a difference map and was refined freely. The remaining hydrogen atoms were included in idealised positions and their Uiso values were set to ride on the Ueq values of the parent carbon atoms. At the conclusion of the refinement, wR_2 = 0.114 and R_1 = 0.047 (2B) for all 4,403 reflections weighted $w = [\sigma^2(F_o^2) + (0.00622\ P)^2 + 23.187\ P]^{-1}$ with $P = (F_o^2 + 2F_c^2)/3$; for the 'observed' data only, R_1 = 0.043.

In the final difference map, the highest peak (*ca* 1.6 eÅ^{-3}) was near Cl(24).

Scattering factors for neutral atoms were taken from reference (3). Computer programs used in this analysis have been noted above, and were run through WinGX (4) on a Dell Optiplex 780 PC at the University of East Anglia.

References

(1) Programs CrysAlisPro, Rigaku Oxford Diffraction Ltd., Abingdon, UK (2023).

(2) G. M. Sheldrick, Programs for crystal structure determination (SHELXT), *Acta Cryst.* (2015) A**71**, 3-8, and refinement (SHELXL), *Acta Cryst.* (2008) A**64**, 112-122 and (2015) C**71**, 3-8.

(3) *'International Tables for X-ray Crystallography'*, Kluwer Academic Publishers,

Dordrecht (1992). Vol. C, pp. 500, 219 and 193.

(4) L. J. Farrugia, *J. Appl. Cryst.* (2012) **45**, 849–854.

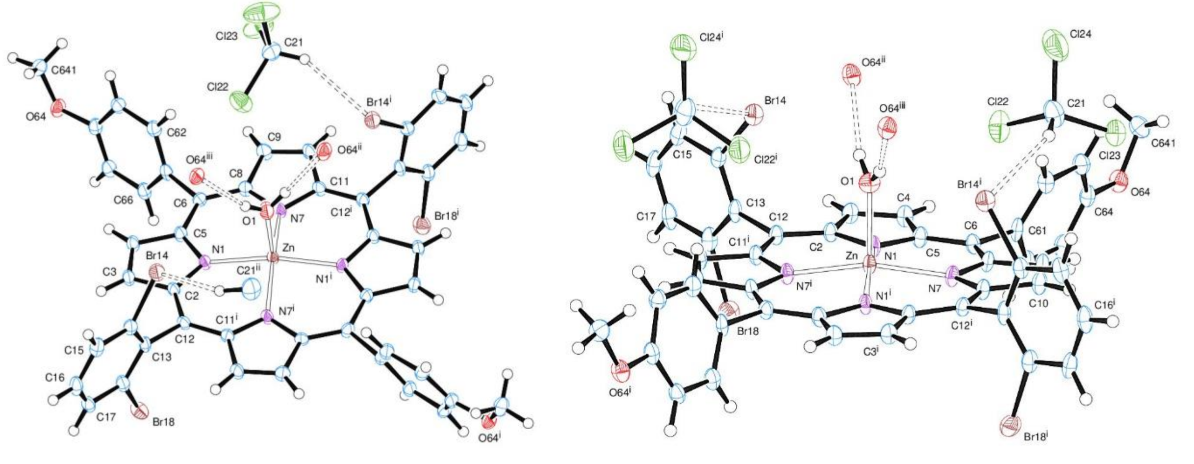

Figure 1. Two views of a molecule of the Zn-O-porphyrin derivative, indicating the atom numbering scheme. Thermal ellipsoids are drawn at the 50% probability level.

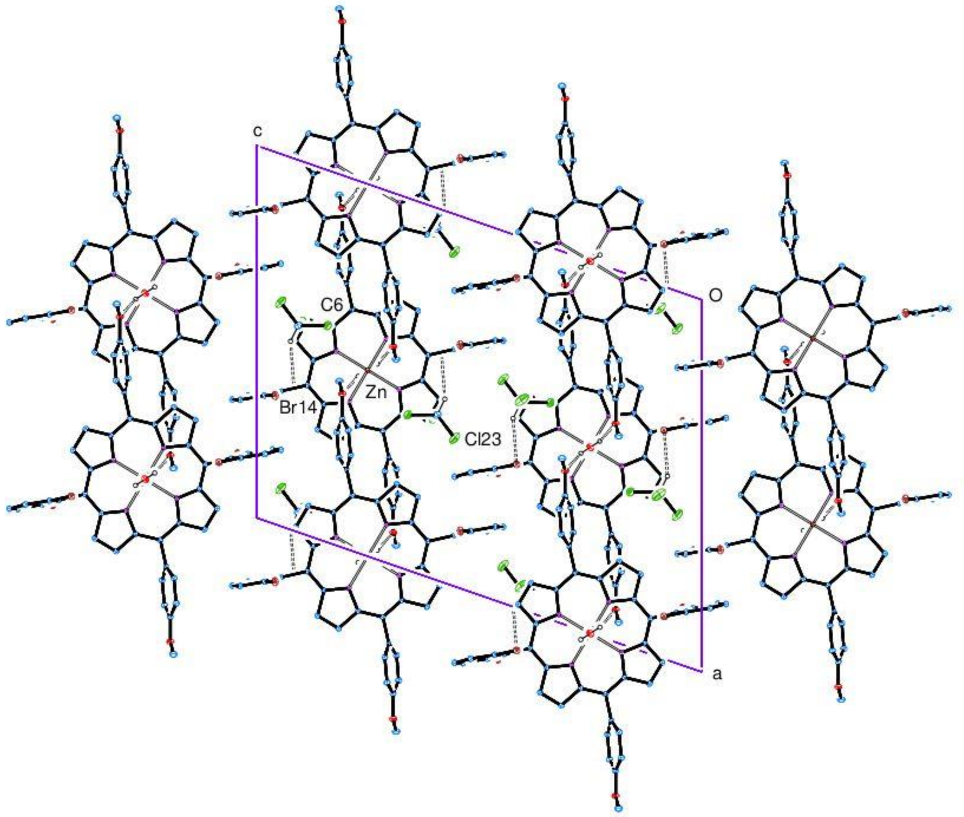

Figure 2. A sheet of hydrogen-bonded molecules in the *a*-*b* plane. The dibromo-phenyl groups are directed out of this plane and have been omitted for clarity.

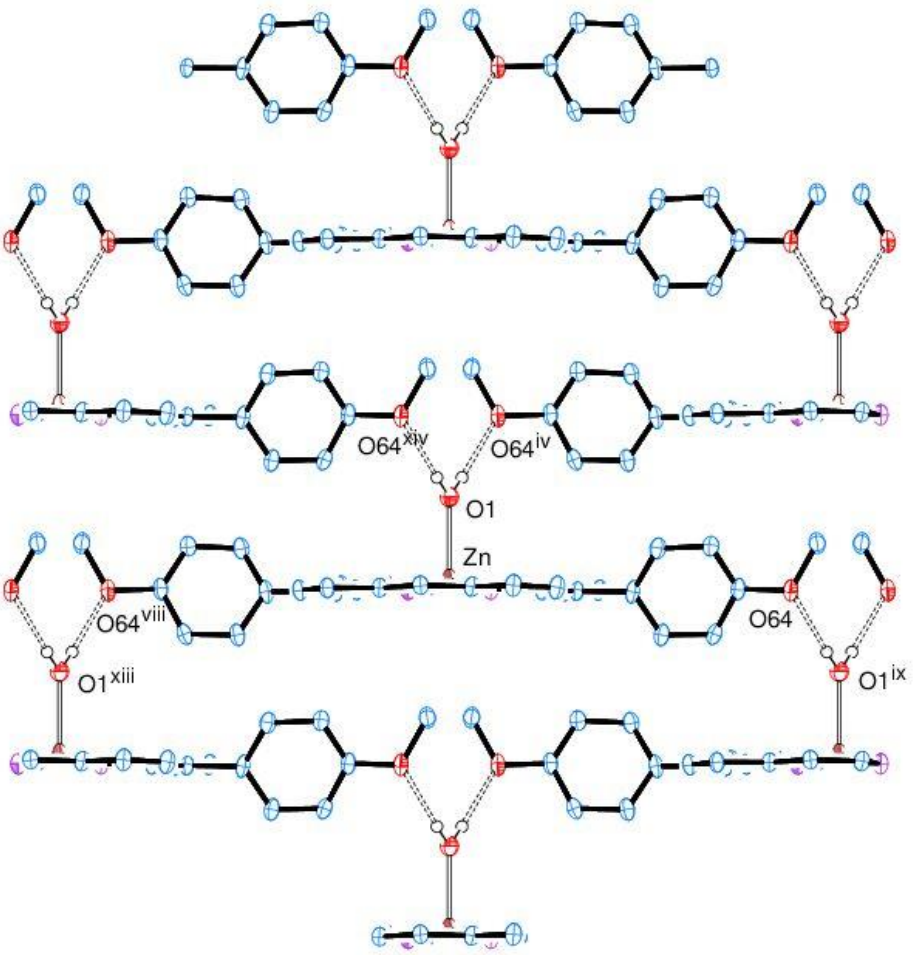

Figure 3. The hydrogen-bonded sheet of Figure 2 rotated about the horizontal, *a*, axis, i.e. this is a view along the *b* axis. The dibromo-phenyl groups are directed out of the sheet and have been omitted for clarity.

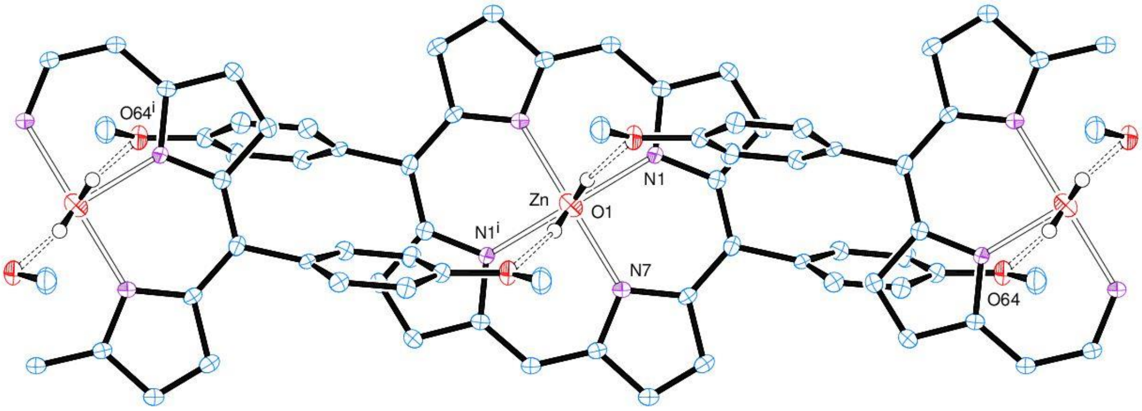

Figure 4. View of the packing of molecules, along the *b* axis.

Notes on the structure

The zinc complex molecule shows a square pyramidal coordination pattern about the zinc atom, Figure 1. The zinc and oxygen atoms lie on a twofold symmetry axis. The zinc atom lies 0.287(2) Å from the mean-plane of the four coordinating N atoms.

The methine hydrogen atoms of the solvent molecules are hydrogen bonded to the Br(14) atoms.

The two (symmetry related) water [of O(1)] hydrogen atoms form good hydrogen bonds with the O(64) atoms of neighbouring molecules, thus linking the molecules in sheets parallel to the *a*-*b* plane, Figures 2 and 3.

C(15) lies over the centrosymmetrically related C(15') where the two phenyl rings overlap with an interplanar distance of 3.36 Å.
